# Sustainable synthesis of 3-substituted phthalides via a catalytic one-pot cascade strategy from 2-formylbenzoic acid with β-keto acids in glycerol

**DOI:** 10.3762/bjoc.13.139

**Published:** 2017-07-19

**Authors:** Lina Jia, Fuzhong Han

**Affiliations:** 1 College of Chemistry and Chemical Engineering, Qiqihar University, Qiqihar 161006, China

**Keywords:** β-keto acid, decarboxylation, glycerol, one-pot cascade reaction, phthalide

## Abstract

**Background:** Phthalides are privileged constituents of numerous pharmaceuticals, natural products and agrochemicals and exhibit several biological and therapeutic activities. Therefore, the development of new, facile, and sustainable strategies for the construction of these moieties is highly desired.

**Results:** A broad substrate scope for β-keto acids was found to be strongly compatible with this catalytic process, affording a wide variety of 3-substituted phthalides in good to excellent yields.

**Conclusion:** A concise and efficient synthesis strategy of 3-substituted phthalides from 2-formylbenzoic acid and β-keto acids via a catalytic one-pot cascade reaction in glycerol has been accomplished.

## Introduction

The phthalides, also known as isobenzofuran-1(3*H*)-ones, are an important class of heterocycles which are of continued interest for chemists [1–2]. 3-Substituted phthalides, which are recognized as versatile building blocks found in a large number of bioactive compounds [3–5], exhibit a broad spectrum of pharmacological activity, such as antibacterial, anti-HIV, antifungal, antibiotic, antitumor and immunosuppressive effects [6–10]. In addition, the one-pot cascade transformation has proven to be the key step in organic syntheses because of its broad functional group compatibility and ingenious construction of molecular architectures [11–14]. Consequently, an efficient synthesis of 3-substituted phthalides via a one-pot cascade reaction is still in high demand.

An analogous approach was described involving a catalytic sequence of the aldol/cyclization reaction of 2-formylbenzoic acid and substituted ketones catalyzed by strong acids [15–17], strong bases [18–19] and solid acid catalysts [20–21]. The majority of these protocols generally suffer from one or more drawbacks, such as the requirement of stoichiometric or excess amounts of strong, corrosive and harmful acids or bases as catalyst, harsh conditions and low functional group tolerance when substituted ketones are used as nucleophiles. Therefore, the importance of phthalides and the increasing awareness of the need for environmentally benign chemical production provide the demand and potential for the development of a green one-pot cascade aldol/cyclization strategy for these compounds [22].

**Scheme 1:**
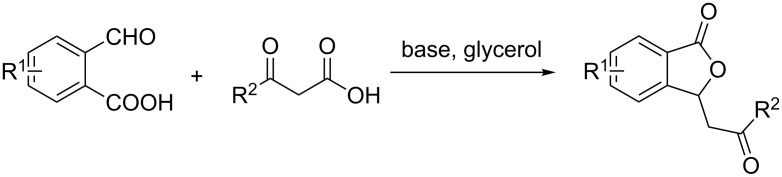
Synthesis of 3-substituted phthalides from substituted 2-formylbenzoic acids and β-keto acids.

The use of β-keto acids as ketone enolate equivalents in metal- and organocatalytic decarboxylative aldol reactions has been extensively studied and proven to be a valuable and straightforward method for the preparation of several biologically active compounds of medicinal and agrochemical interest [23–30]. Notably, the decarboxylative reaction of β-keto acids provides a traceless means of activation with CO_2_ as the only byproduct. On the other hand, glycerol, as an environmentally friendly reaction medium, has received increasing interest for organic reactions because of its peculiar physical and chemical properties such as polarity, low toxicity, biodegradability, high boiling point, and ready availability from renewable feed stocks [31]. Many organic transformations have been accomplished using glycerol as a solvent [32–40]. Therefore, as part of our ongoing research interest in exploring novel and sustainable transformations of β-keto acids [41–42], we envisioned that the direct utilization of β-keto acids as nucleophilic species and glycerol as a solvent will allow the establishment of an efficient catalytic one-pot cascade aldol/cyclization reaction. Herein, we present a new method to provide a convenient, green and efficient alternative for rapidly accessing a variety of 3-substituted phthalides (Scheme 1).

## Results and Discussion

To optimize the reaction conditions, 2-formylbenzoic acid (**1a**) and benzoylacetic acid (**2a**) were chosen as model substrates. As summarized in Table 1, the reaction did not occur without the use of a base, indicating that a promoter is essential for an effective transformation (Table 1, entry 1). We then turned our attention to the examination of a series of base catalysts for the present annulation protocol. To our delight, the reaction proceeded smoothly to give the desired 3-phenacylphthalide (**3a**) in 32% yield with Et_3_N (20 mol %) as a catalyst at 65 °C (Table 1, entry 2). To improve the efficiency of the reaction, a detailed optimization study was performed with various bases, such as tertiary, secondary and primary amines. When tertiary amines were used as the catalyst, very low yields of **3a** were obtained (Table 1, entries 3–5). Unfortunately, a sluggish reaction was observed in the presence of pyrrolidine (Table 1, entry 6). Gratifyingly, the desired product was obtained in the presence of primary amines in good yields (Table 1, entries 7–9). In addition, the use of several inorganic bases delivered no catalytic activation (Table 1, entries 10 and 11). Notably, a higher reaction temperature did not increase the yield of **3a** (Table 1, entry 12). Overall, the best result was achieved when 20 mol % of *p*-anisidine was used as the catalyst in glycerol at 65 °C for 0.5 h, providing **3a** in 80% yield (Table 1, entry 9).

**Table 1:** Optimization of the reaction conditions^a^.

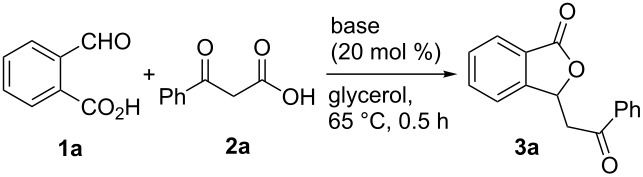		

Entry	Catalyst	Yield (%)^b^

1	none	–^c^
2	Et_3_N	32
3	iPr_2_NEt	11
4	*N*-methylmorpholine	trace
5	DMAP	–^c^
6	pyrrolidine	trace
7	benzylamine	56
8	aniline	67
9	*p*-anisidine	80
10	K_2_CO_3_	–^c^
11	NaOH	–^c^
12	*p*-anisidine	80^d^

^a^General reaction conditions: **1a** (0.5 mmol), **2a** (1.0 mmol) and base (0.1 mmol) in glycerol (3 mL) at 65 °C for 0.5 h. DMAP = 4-dimethylaminopyridine. ^b^The yields indicated are the isolated yields after column chromatography. ^c^No reaction. ^d^The reaction was carried out at 90 °C.

**Scheme 2:**
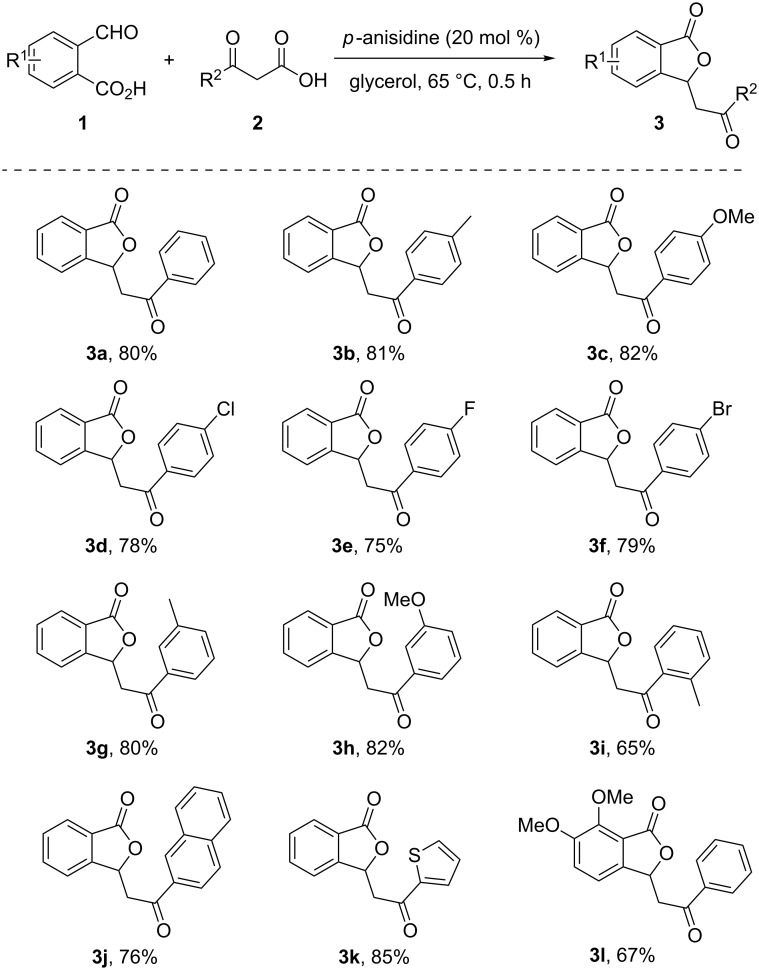
Substrate scope of the synthesis of 3-substituted phthalides. General reaction conditions: **1** (0.5 mmol), **2** (1.0 mmol) and *p*-anisidine (0.1 mmol) in glycerol (3 mL) at 65 °C for 0.5 h. The yields indicated are the isolated yields after column chromatography.

With the optimal conditions in hand, we started to explore the scope and limitations of this one-pot cascade aldol/cyclization system. Typical results are shown in Scheme 2. The annulation proceeded smoothly with β-keto acids **2** bearing diverse arene substituents to provide the corresponding isobenzofuran-1(3*H*)-ones in moderate to excellent yields. For *para*-substituted β-keto acids **2b–f**, both electron-donating and electron-withdrawing groups such as methyl (**2b**), methoxy (**2c**), and halogens (**2d–f**) afforded the products in relatively high reaction yields. The use of *meta*-aryl substituted β-keto acids provided the products in good yields, whereas β-keto acid **2i**, bearing a methyl group at the *ortho*-position, was evaluated to produce the desired compound **3i** in moderate yield due to steric hindrance. Moreover, 3-(naphthalen-2-yl)-3-oxopropanoic acid (**2j**) turned out to be a good substrate, and the corresponding product was obtained in 76% yield. It is worth mentioning that even a β-keto acid bearing a heteroaromatic ring (**2k**) afforded the desired product in 85% yield. Unfortunately, acetoacetic acid did not undergo the reaction due to its low reactivity. Finally, the substituted 2-formylbenzoic acid **1b** (5,6-(OCH_3_)_2_) was also tested for this transformation, and the target product **3l** was obtained with 67% yield.

After investigating the scope of the synthesis of 3-substituted phthalides, the recyclability of glycerol was investigated for the reaction shown in Table 2. After completion of the condensation, the mixture was extracted with ethyl acetate. The glycerol phase was directly reused for further reactions. We were pleased to observe that the yield of **3a** was almost consistent after four runs.

**Table 2:** Reuse of glycerol in the synthesis of **3a**.

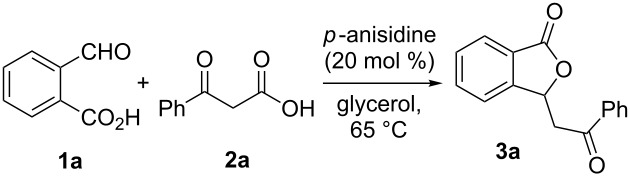		

Run	Reaction time (h)	Yield (%)^a^

1^b^	0.5	80
2^c^	0.5	80
3^c^	1.0	80
4^c^	1.0	78

^a^The yields indicated are the isolated yields after column chromatography. ^b^General reaction conditions: **1** (0.5 mmol), **2a** (1.0 mmol) and *p*-anisidine (0.1 mmol) in glycerol (3 mL) at 65 °C for 0.5 h. ^c^Recovered glycerol was used.

**Scheme 3:**
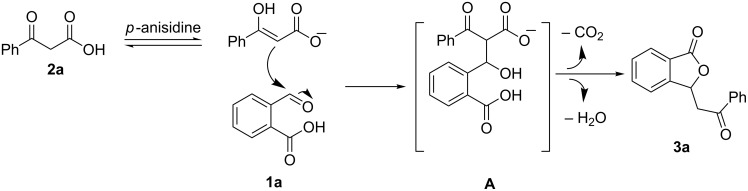
Possible mechanistic pathway.

On the basis of the results described above and other previous work [29,43], a plausible mechanism for this reaction has been tentatively proposed (Scheme 3). Firstly, 2-formylbenzoic acid (**1a**) is attacked preferably by benzoylacetic acid (**2a**) in the presence of a base to afford the aldol intermediate **A**. Next, the subsequent facile decarboxylation and lactonization of intermediate **A** leads to 3-phenacylphthalide (**3a**).

## Conclusion

In summary, we have accomplished a sustainable and efﬁcient one-pot cascade aldol/cyclization methodology using the low-cost and renewable feedstock glycerol as the solvent. A broad substrate scope for β-keto acids is strongly compatible with this catalytic process, affording a wide variety of 3-substituted phthalides in good to excellent yields. Further efforts on the development of other analogues of this transformation are currently underway in our laboratory.

## Supporting Information

File 1 Experimental procedures, characterization data and copies of NMR spectra.
