# Supplementary material for: Sustainable synthesis of 3-substituted phthalides via a catalytic one-pot cascade strategy from 2-formylbenzoic acid with β-keto acids in glycerol
Source: Beilstein J Org Chem. 2017 Jul 19;13:1425–9. doi: 10.3762/bjoc.13.139 (PMC5530723; doi:10.3762/bjoc.13.139)
Supplement: File 1 — Experimental procedures, characterization data and copies of NMR spectra. [file Beilstein_J_Org_Chem-13-1425-s001.pdf]

Supporting Information

for

Sustainable synthesis of 3-substituted phthalides via a catalytic one-pot cascade strategy from 2-formylbenzoic acid with β-keto acids in glycerol

Lina Jia* and Fuzhong Han

Address: College of Chemistry and Chemical Engineering, Qiqihar University, Qiqihar 161006, China

Email: Lina Jia - jialinachn@163.com

*Corresponding author

Experimental procedures, characterization data and copies of NMR spectra

Table of Contents

General information……………………………………………………………………………….S2

Preparation of β-keto acids……………………………………………………………………….S2

Typical procedure for the one-pot cascade reaction……………………………………………..S2

References………………………………………………………………………………………..S5

NMR spectra……………………………………………………………………………………..S6

General information

All reactions were carried out under air. Solvents were purified by standard procedure before use. Commercial solvents were used without further purification. Flash chromatography was performed on silica gel 60 (40–63 μm, 60 Å). Thin layer chromatography (TLC) was performed on glass plates coated with silica gel 60 with F254 indicator. Electrospray ionization high-resolution mass spectra (ESI-HRMS) were recorded on a Bruker P-SIMS-Gly FT-ICR mass spectrometer. Proton nuclear magnetic resonance (^{1}H NMR) spectra were recorded on a Bruker 600 MHz spectrometer. Chemical shifts for protons are reported in parts per million downfield from tetramethylsilane and are referenced to residual protium in the NMR solvent ($CHCl_3$, δ 7.26). Carbon nuclear magnetic resonance (^{13}C NMR) spectra were recorded on a Bruker 150 MHz spectrometer. Chemical shifts for carbon are reported in parts per million downfield from tetramethylsilane and are referenced to the carbon resonances of the solvent ($CDCl_3$, δ 77.07). Data are represented as follows: chemical shift, multiplicity (br = broad, s = singlet, d = doublet, t = triplet, q = quartet, m = multiplet), coupling constants in Hertz (Hz), integration.

Preparation of β-keto acids[1]

To a β-keto ester (10 mmol) at room temperature was added aqueous sodium hydroxide (1 N, 20 mL). The mixture was stirred vigorously at room temperature for 12 h, and extracted with CH_2Cl_2 (2 × 20 mL). The aqueous layer was cooled with ice-water and acidified with aqueous HCl (1 N) until pH ≈ 2, and extracted with CH_2Cl_2 (2 × 60 mL). The combined organic layers were dried over anhydrous sodium sulfate, and evaporated under reduced pressure at a temperature below 30 °C to give β-keto acid.

Typical procedure for the one-pot cascade reaction

In 25 mL Schlenk flask were placed **1** (0.5 mmol), **2** (1.0 mmol), $PMPNH_2$ (0.2 mmol, 20 mol %), and 3.0 mL glycerol. The mixture was heated at 65 °C for 0.5 h, cooled, and extracted three times with ethyl acetate. After concentration under reduced pressure, the resulting residue was purified by column chromatography (SiO_2; hexanes/EtOAc 4:1) to give product **3**.

3-(2-Oxo-2-phenylethyl)isobenzofuran-1(3*H*)-one (3a).[2] White solid, 80% yield, m.p. = 143-145 ^{o}C. ^{1}H NMR (600 MHz, $CDCl_3$) δ 7.97 (d, *J* = 7.9 Hz, 2H), 7.93 (d, *J* = 7.6 Hz, 1H), 7.67 (t, *J* = 7.5 Hz, 1H), 7.62 (t, *J* = 7.4 Hz, 1H), 7.59-7.54 (m, 2H), 7.50 (t, *J* = 7.7 Hz, 2H), 6.19 (t, *J* = 6.5 Hz, 1H), 3.79 (dd, *J* = 17.6, 5.7 Hz, 1H), 3.40 (dd, *J* = 17.6, 7.4 Hz, 1H). ^{13}C NMR (151 MHz, $CDCl_3$) δ 196.1, 170.2, 149.8

136.2, 134.3, 133.9, 129.5, 128.9, 128.2, 125.9, 125.8, 122.9, 77.2, 43.7.

3-(2-Oxo-2-(*p*-tolyl)ethyl)isobenzofuran-1(3*H*)-one (3b).[2] White solid, 81% yield, m.p. = 148-149 °C. ^{1}H NMR (600 MHz, $CDCl_3$) δ 7.92 (d, *J* = 7.7 Hz, 1H), 7.86 (d, *J* = 8.2 Hz, 2H), 7.66 (td, *J* = 7.7, 1.0 Hz, 1H), 7.58- 7.53 (m, 2H), 7.28 (d, *J* = 7.9 Hz, 2H), 6.18 (t, *J* = 6.6 Hz, 1H), 3.76 (dd, *J* = 17.5, 5.7 Hz, 1H), 3.36 (dd, *J* = 17.5, 7.5 Hz, 1H), 2.42 (s, 3H). ^{13}C NMR (151 MHz, $CDCl_3$) δ 195.67, 170.2, 149.9, 144.9, 134.3, 133.8, 129.5, 129.4, 128.3, 125.9, 125.8, 122.9, 77.3, 43.6, 21.7.

3-(2-(4-Methoxyphenyl)-2-oxoethyl)isobenzofuran-1(3*H*)-one (3c).[2] White solid, 82% yield, m.p. = 116-117°C. ^{1}H NMR (600 MHz, DMSO) δ 7.99 (d, *J* = 8.9 Hz, 2H), 7.86 (d, *J* = 7.6 Hz, 1H), 7.79 (t, *J* = 7.4 Hz, 1H), 7.74 (d, *J* = 7.6 Hz, 1H), 7.61 (t, *J* = 7.4 Hz, 1H), 7.05 (d, *J* = 8.9 Hz, 2H), 6.11 (dd, *J* = 7.8, 4.3 Hz, 1H), 3.85 (s, 3H), 3.75 (dd, *J* = 17.8, 4.3 Hz, 1H), 3.68 (dd, *J* = 17.8, 8.0 Hz, 1H). ^{13}C NMR (151 MHz, DMSO) δ 195.2, 170.3, 164.0, 150.5, 134.8, 131.1, 129.7, 129.7, 126.0, 125.4, 123.4, 114.5, 77.8, 56.1, 42.9.

3-(2-(4-Chlorophenyl)-2-oxoethyl)isobenzofuran-1(3*H*)-one (3d).[2] White solid, 78% yield, m.p. = 145-146 °C. ^{1}H NMR (600 MHz, $CDCl_3$) δ 7.93-7.90 (m, 3H), 7.70-7.66 (m, 1H), 7.56 (t, *J* = 6.7 Hz, 2H), 7.47 (d, *J* = 8.6 Hz, 2H), 6.16 (t, *J* = 6.5 Hz, 1H), 3.73 (dd, *J* = 17.6, 6.0 Hz, 1H), 3.37 (dd, *J* = 17.6, 7.1 Hz, 1H). ^{13}C NMR (151 MHz, $CDCl_3$) δ 194.8, 170.0, 149.6, 140.5, 134.5, 134.3, 129.6, 129.6, 129.2, 125.9, 125.8, 122.7, 77.0, 43.7.

3-(2-(4-Fluorophenyl)-2-oxoethyl)isobenzofuran-1(3*H*)-one (3e).[2] White solid, 75% yield, m.p. = 134-135 °C. ^{1}H NMR (600 MHz, DMSO) δ 8.10 (dd, *J* = 8.7, 5.6 Hz, 2H), 7.86 (d, *J* = 7.6 Hz, 1H), 7.80 (t, *J* = 7.4 Hz, 1H), 7.74 (d, *J* = 7.6 Hz, 1H), 7.62 (t, *J* = 7.4 Hz, 1H), 7.37 (t, *J* = 8.8 Hz, 2H), 6.12 (dd, *J* = 7.9, 4.1 Hz, 1H), 3.85 (dd, *J* = 18.0, 4.1 Hz, 1H), 3.74 (dd, *J* = 18.0, 8.0 Hz, 1H). ^{13}C NMR (151 MHz, DMSO) δ 195.6, 170.2, 166.6, 164.9, 150.4, 134.8, 133.5 (d, *J* = 2.5 Hz), 131.7 (d, *J* = 9.6 Hz), 129.8, 126.0, 125.4, 123.4, 116.3 (d, *J* = 21.9 Hz), 77.6, 43.2.

3-(2-(4-Bromophenyl)-2-oxoethyl)isobenzofuran-1(3*H*)-one (3f).[2] White solid, 79% yield, m.p. = 147-148 °C. ^{1}H NMR (600 MHz, $CDCl_3$) δ 7.92 (d, *J* = 7.8 Hz, 1H), 7.82 (d, *J* = 8.5 Hz, 2H), 7.67 (t, *J* = 7.5 Hz, 1H), 7.63 (d, *J* = 8.5 Hz, 2H), 7.55 (dd, *J* = 7.3, 4.8 Hz, 2H), 6.16 (t, *J* = 6.5 Hz, 1H), 3.72 (dd, *J* = 17.6, 6.0 Hz, 1H), 3.37 (dd, *J* = 17.6, 7.0 Hz, 1H). ^{13}C NMR (151 MHz, $CDCl_3$) δ 195.0, 170.0, 149.5, 134.9, 134.4, 132.2, 129.7, 129.6, 129.2, 125.9, 125.9, 122.7, 77.0, 43.7.

3-(2-Oxo-2-(*m*-tolyl)ethyl)isobenzofuran-1(3*H*)-one (3g).[2] White solid, 80% yield, m.p. = 104-105 °C. ^{1}H NMR (600 MHz, $CDCl_3$) δ 7.92 (d, *J* = 7.6 Hz, 1H), 7.78 (s, 1H), 7.75 (d, *J* = 7.7 Hz, 1H), 7.67-7.65 (m, 1H), 7.58-7.53 (m, 2H), 7.42 (d, *J* = 7.5 Hz, 1H), 7.37 (t, *J* = 7.6 Hz, 1H), 6.22-6.13 (m,

1H), 3.77 (dd, *J* = 17.6, 5.7 Hz, 1H), 3.39 (dd, *J* = 17.6, 7.4 Hz, 1H), 2.42 (s, 3H). ^{13}C NMR (151 MHz, $CDCl_3$) δ 196.3, 170.2, 149.8, 138.7, 136.2, 134.7, 134.3, 129.4, 128.7, 128.7, 125.9, 125.8, 125.4, 122.9, 77.1, 43.8, 21.4.

3-(2-(*m*-Methoxyphenyl)-2-oxoethyl)isobenzofuran-1(3*H*)-one (3h).[2] White solid, 82% yield, m.p. = 116-117°C. ^{1}H NMR (600 MHz, DMSO) δ 7.99 (d, *J* = 8.9 Hz, 2H), 7.86 (d, *J* = 7.6 Hz, 1H), 7.79 (t, *J* = 7.4 Hz, 1H), 7.74 (d, *J* = 7.6 Hz, 1H), 7.61 (t, *J* = 7.4 Hz, 1H), 7.05 (d, *J* = 8.9 Hz, 2H), 6.11 (dd, *J* = 7.8, 4.3 Hz, 1H), 3.85 (s, 3H), 3.75 (dd, *J* = 17.8, 4.3 Hz, 1H), 3.68 (dd, *J* = 17.8, 8.0 Hz, 1H). ^{13}C NMR (151 MHz, DMSO) δ 195.2, 170.3, 164.0, 150.5, 134.8, 131.1, 129.7, 129.7, 126.0, 125.4, 123.4, 114.5, 77.8, 56.1, 42.9.

3-(2-Oxo-2-(o-tolyl)ethyl)isobenzofuran-1(3*H*)-one (3i).[2] White solid, 65% yield, m.p. = 98-99 °C. ^{1}H NMR (600 MHz, $CDCl_3$) δ 7.92 (d, *J* = 7.6 Hz, 1H), 7.68 (t, *J* = 7.5 Hz, 1H), 7.63 (d, *J* = 7.6 Hz, 1H), 7.56 (dd, *J* = 15.2, 7.6 Hz, 2H), 7.41 (t, *J* = 7.4 Hz, 1H), 7.27 (dd, *J* = 16.6, 6.7 Hz, 3H), 6.16 (t, *J* = 6.4 Hz, 1H), 3.67 (dd, *J* = 17.4, 6.3 Hz, 1H), 3.36 (dd, *J* = 17.4, 6.7 Hz, 1H), 2.59 (s, 3H). ^{13}C NMR (151 MHz, $CDCl_3$) δ 199.3, 170.1, 149.8, 139.0, 136.5, 134.3, 132.4, 132.3, 129.5, 129.0, 126.0, 125.9, 125.8, 122.6, 77.4, 46.2, 21.7.

3-(2-(Naphthalen-2-yl)-2-oxoethyl)isobenzofuran-1(3*H*)-one (3j). White solid, 76% yield, m.p. = 161-162 °C. ^{1}H NMR (600 MHz, $CDCl_3$) δ 8.45 (s, 1H), 8.05 (d, *J* = 8.6 Hz, 1H), 7.93 (dd, *J* = 13.5, 7.7 Hz, 3H), 7.89 (d, *J* = 8.1 Hz, 1H), 7.67 (t, *J* = 7.5 Hz, 1H), 7.62 (dd, *J* = 12.6, 7.5 Hz, 2H), 7.56 (dd, *J* = 14.6, 7.4 Hz, 2H), 6.25 (t, *J* = 6.5 Hz, 1H), 3.93 (dd, *J* = 17.4, 5.7 Hz, 1H), 3.54 (dd, *J* = 17.4, 7.4 Hz, 1H). ^{13}C NMR (151 MHz, $CDCl_3$) δ 196.0, 170.2, 149.8, 135.9, 134.3, 133.6, 132.4, 130.3, 129.7, 129.5, 129.0, 128.8, 127.9, 127.1, 126.0, 125.8, 123.5, 122.9, 77.3, 43.8. HRMS calc. for $[M+Na]^+$ $C_{20}H_{14}NaO_3$: 325.0841, found: 325.0851.

3-(2-Oxo-2-(thiophen-2-yl)ethyl)isobenzofuran-1(3*H*)-one (3k).[2] White solid, 85% yield, m.p. = 133-134 °C. ^{1}H NMR (600 MHz, DMSO) δ 8.07 (d, *J* = 4.9 Hz, 1H), 8.02 (d, *J* = 3.7 Hz, 1H), 7.86 (d, *J* = 7.6 Hz, 1H), 7.80 (t, *J* = 7.4 Hz, 1H), 7.75 (d, *J* = 7.6 Hz, 1H), 7.62 (t, *J* = 7.4 Hz, 1H), 7.27-7.25 (m, 1H), 6.10 (dd, *J* = 8.3, 3.9 Hz, 1H), 3.77 (dd, *J* = 17.3, 4.0 Hz, 1H), 3.68 (dd, *J* = 17.3, 8.4 Hz, 1H). ^{13}C NMR (151 MHz, DMSO) δ 189.8, 170.2, 150.2, 143.8, 136.2, 134.9, 134.8, 129.8, 129.4, 125.9, 125.4, 123.4, 77.6, 43.4.

6,7-Dimethoxy-3-(2-oxo-2-phenylethyl)isobenzofuran-1(3*H*)-one (3l). White solid, 67% yield, m.p. = 129-130 °C. ^{1}H NMR (600 MHz, $CDCl_3$) δ 7.95 (dd, *J* = 8.4, 1.2 Hz, 2H), 7.60 (d, *J* = 7.4 Hz, 1H), 7.49 (dd, *J* = 11.1, 4.6 Hz, 2H), 7.17 (dt, *J* = 8.3, 4.4 Hz, 2H), 6.05 (dd, *J* = 6.9, 6.1 Hz, 1H), 4.11 (s, 3H), 3.90

(s, 3H), 3.74 (dd, *J* = 17.5, 5.7 Hz, 1H), 3.35 (dd, *J* = 17.5, 7.3 Hz, 1H). ^{13}C NMR (151 MHz, $CDCl_3$) δ 196.3, 167.6, 152.8, 148.4, 142.7, 136.3, 133.8, 128.9, 128.2, 119.5, 118.2, 117.3, 75.9, 62.4, 56.9, 44.1. HRMS calc. for [M+H] $C_{18}H_{17}O_5$: 313.1076, found: 313.1071.

References

[1] D. A. Evans, S. Mito, and D. Seidel, *J. Am. Chem. Soc.*, 2007, **129**, 11583.

[2] F. Rastegari, I. Mohammadpoor-Baltork, A. R. Khosropour, Tangestaninejad S., V. Mirkhani, and M. Moghadam, *RSC Adv.*, 2015, **5**, 15274.

NMR Spectra

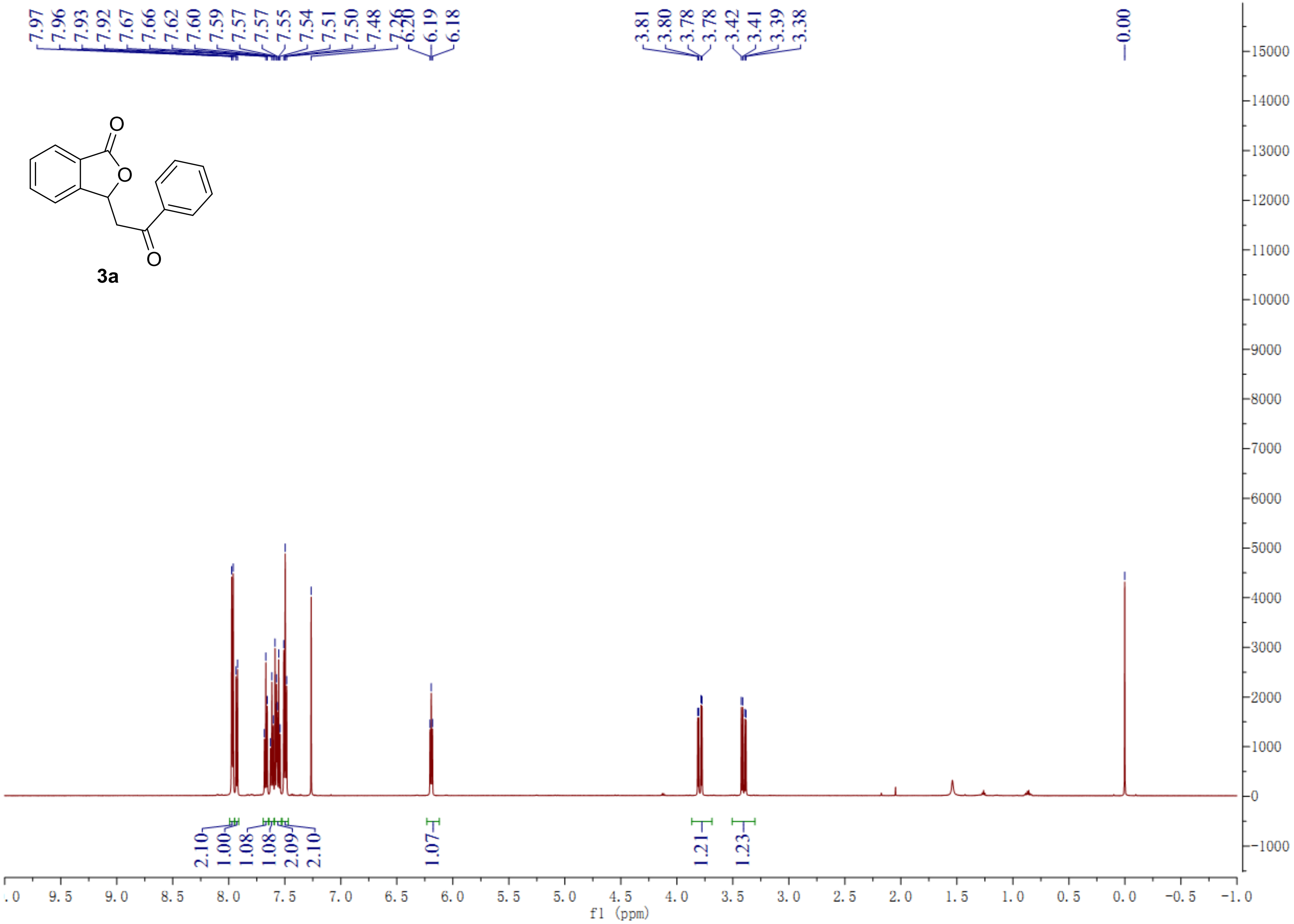
7.97
7.96
7.93
7.92
7.67
7.66
7.62
7.60
7.59
7.57
7.57
7.55
7.54
7.51
7.50
7.48
7.26
6.20
6.19
6.18
3.81
3.80
3.78
3.78
3.42
3.41
3.39
3.38
0.00
3a
2.10
1.00
1.08
1.08
2.09
2.10
1.07
1.21
1.23
f1 (ppm)

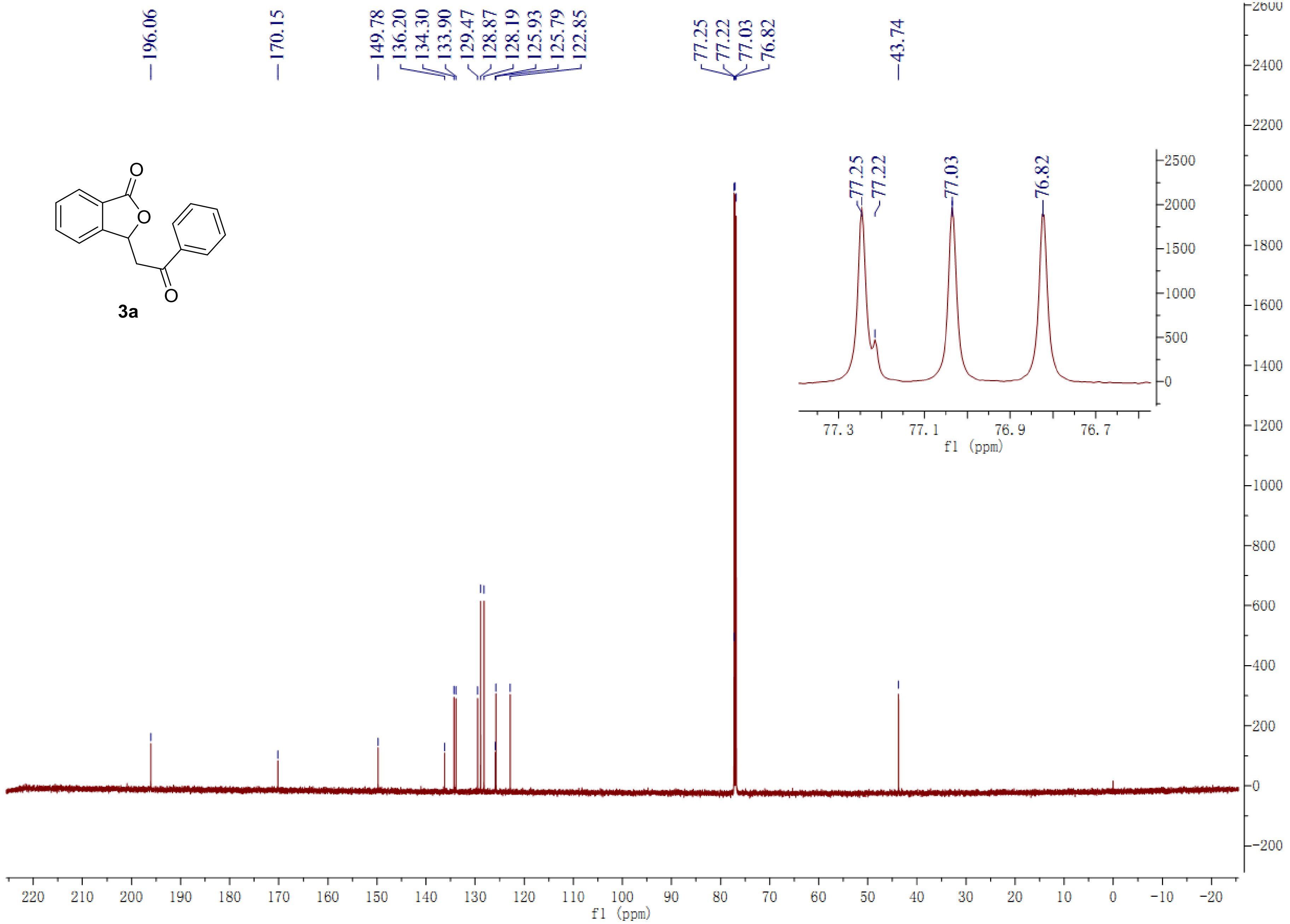

196.06
170.15
149.78
136.20
134.30
133.90
129.47
128.87
128.19
125.93
125.79
122.85
77.25
77.22
77.03
76.82
43.74
3a
f1 (ppm)

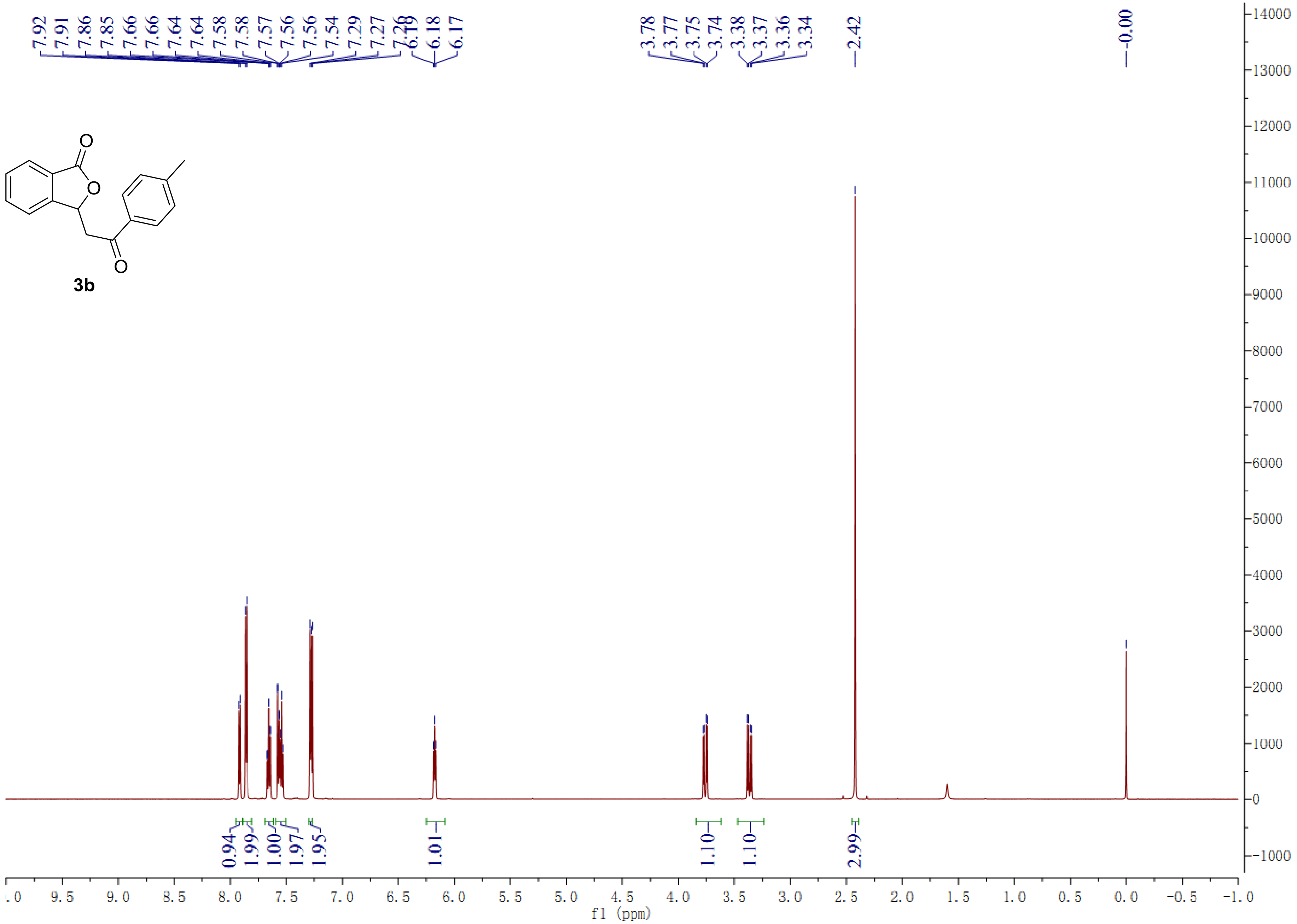
7.92 7.91 7.86 7.85 7.66 7.66 7.64 7.64 7.58 7.58 7.57 7.56 7.56 7.54 7.29 7.27 7.26 6.19 6.18 6.17
3.78 3.77 3.75 3.74 3.38 3.37 3.36 3.34
2.42
0.00
3b
0.94 1.99 1.00 1.97 1.95 1.01 1.10 1.10 2.99
f1 (ppm)

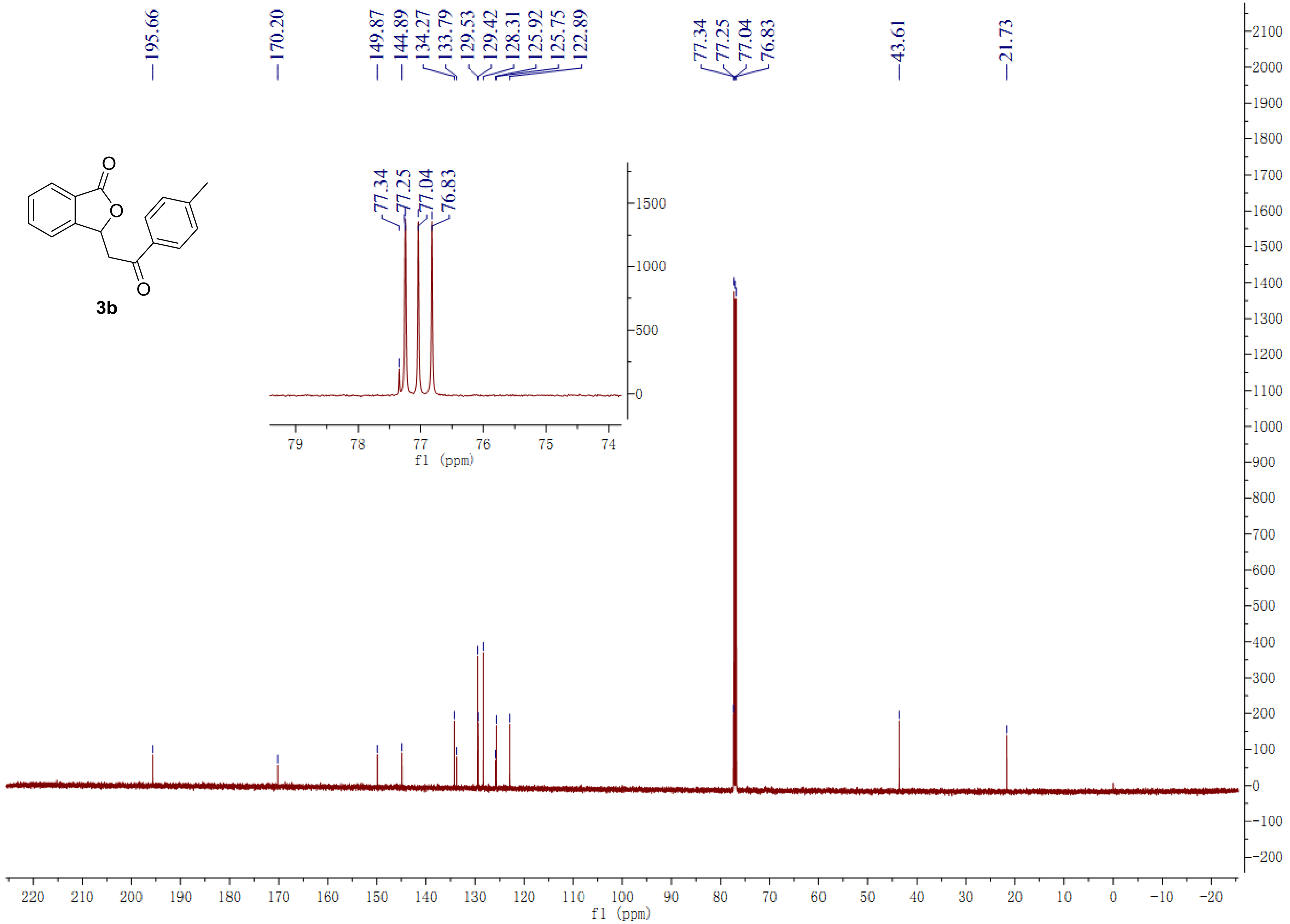
195.66
170.20
149.87
144.89
134.27
133.79
129.53
129.42
128.31
125.92
125.75
122.89
77.34
77.25
77.04
76.83
43.61
21.73
3b
f1 (ppm)

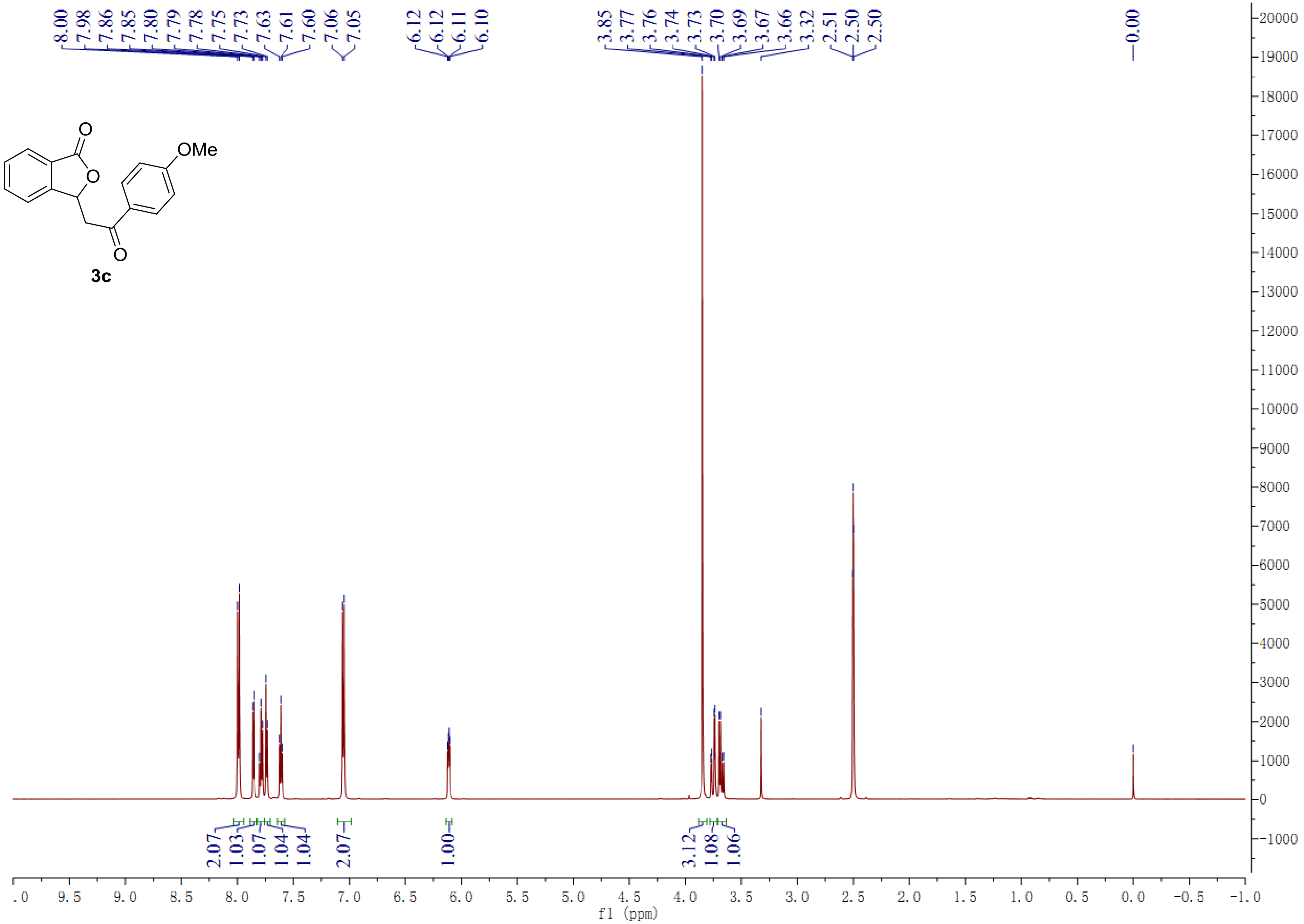

8.00
7.98
7.86
7.85
7.80
7.79
7.78
7.75
7.73
7.63
7.61
7.60
7.06
7.05
6.12
6.12
6.11
6.10
3.85
3.77
3.76
3.74
3.73
3.70
3.69
3.67
3.66
3.32
2.51
2.50
2.50
0.00
O
O
OMe
O
3c
2.07
1.03
1.07
1.04
1.04
2.07
1.00
3.12
1.08
1.06
f1 (ppm)

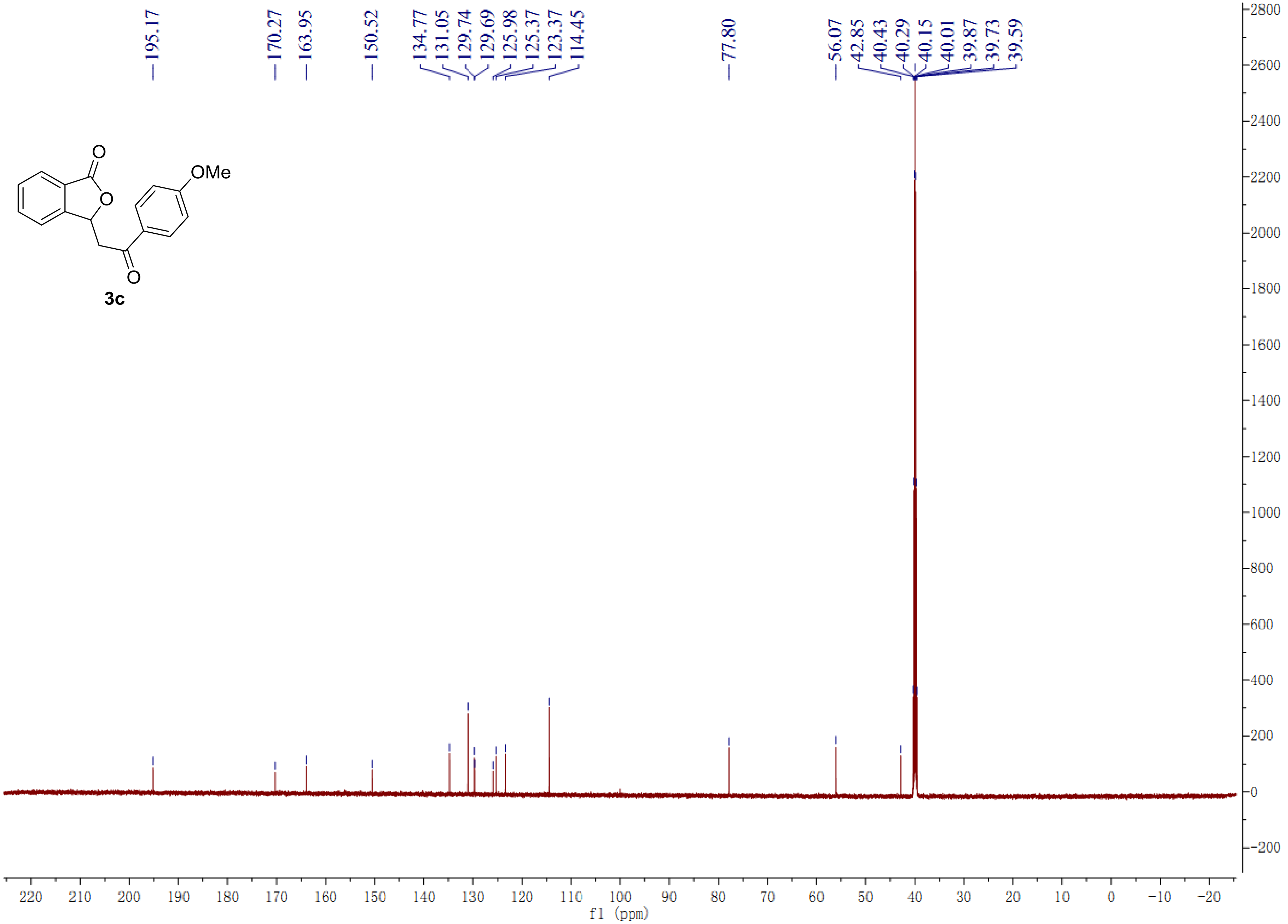
195.17
170.27
163.95
150.52
134.77
131.05
129.74
129.69
125.98
125.37
123.37
114.45
77.80
56.07
42.85
40.43
40.29
40.15
40.01
39.87
39.73
39.59
OMe
O
O
O
f1 (ppm)

3c

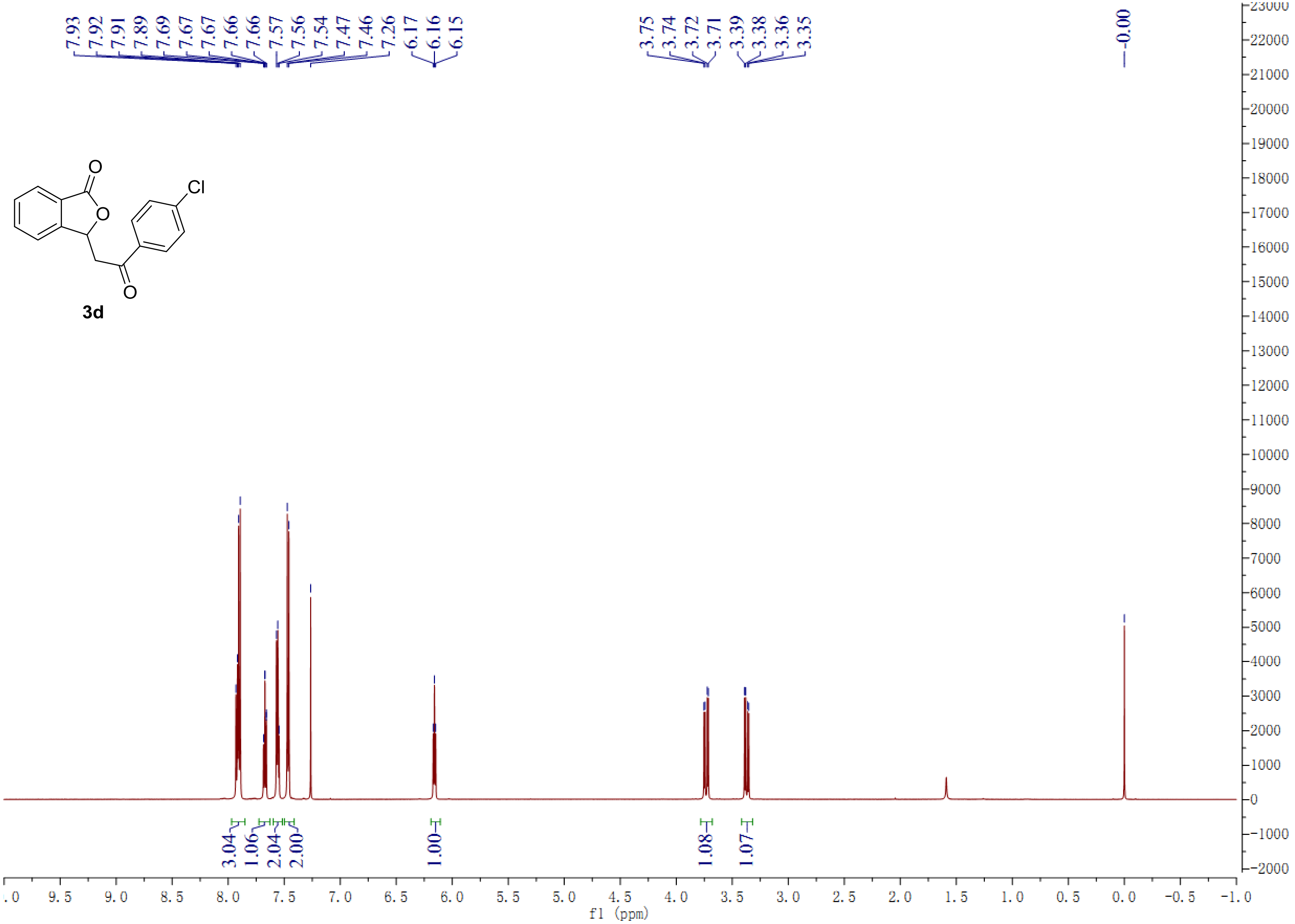
7.93 7.92 7.91 7.89 7.69 7.67 7.67 7.66 7.66 7.57 7.56 7.54 7.47 7.46 7.26 6.17 6.16 6.15
3.75 3.74 3.72 3.71 3.39 3.38 3.36 3.35
-0.00
O
O
Cl
O
3d
3.04
1.06
2.04
2.00
1.00
1.08
1.07
f1 (ppm)

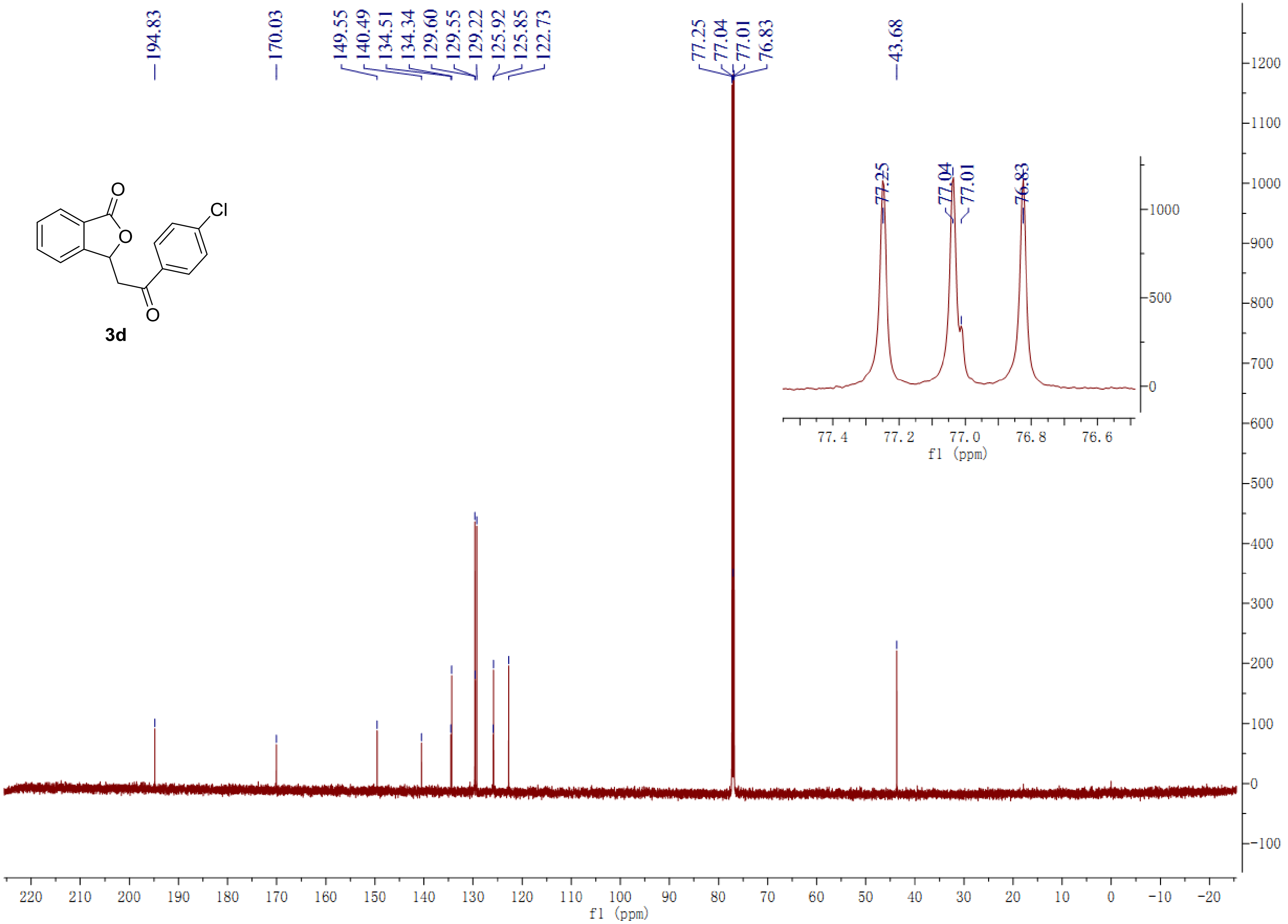
3d
194.83
170.03
149.55
140.49
134.51
134.34
129.60
129.55
129.22
125.92
125.85
122.73
77.25
77.04
77.01
76.83
43.68
f1 (ppm)

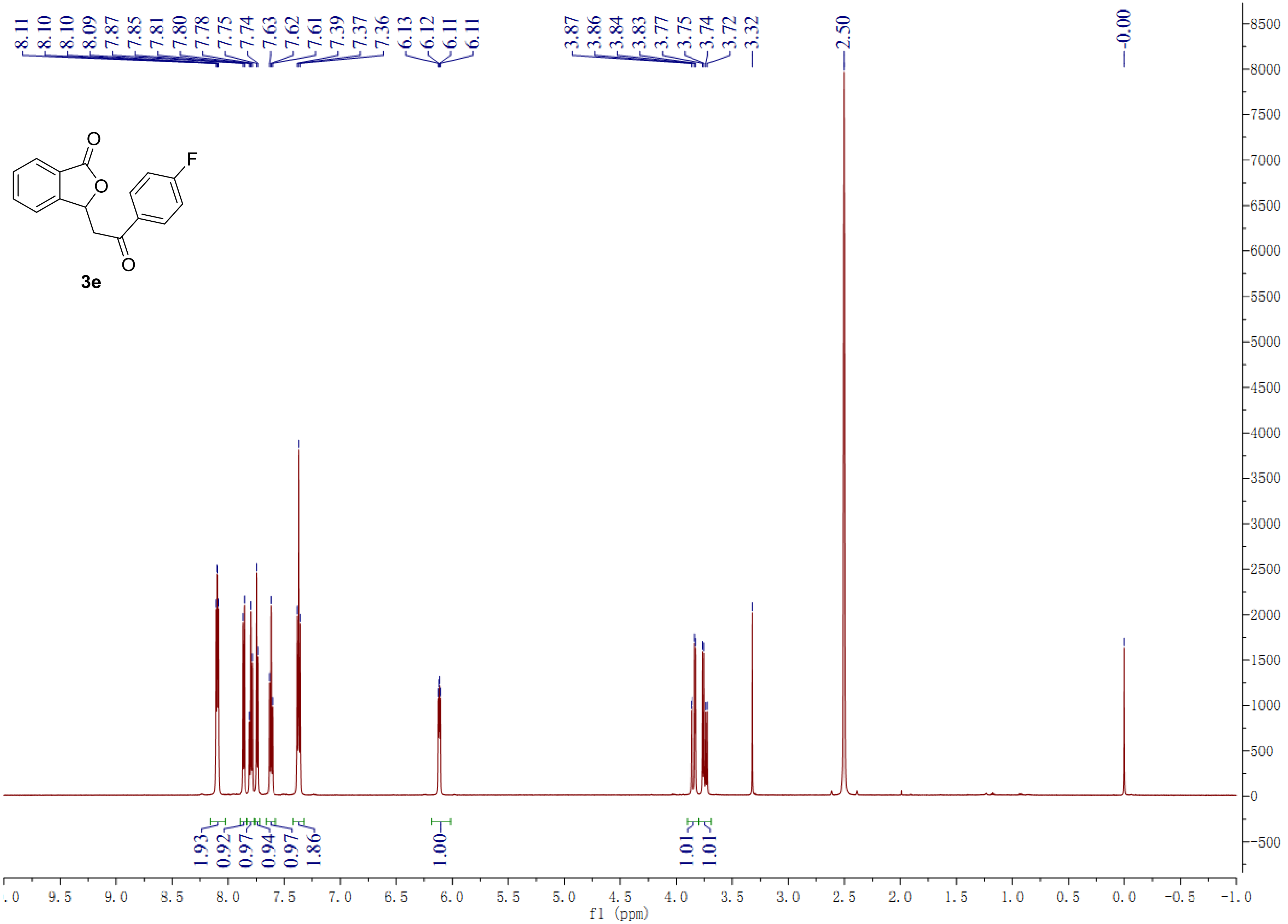
8.11 8.10 8.10 8.09 7.87 7.85 7.81 7.80 7.78 7.75 7.74 7.63 7.62 7.61 7.39 7.37 7.36 6.13 6.12 6.11 6.11
3.87 3.86 3.84 3.83 3.77 3.75 3.74 3.72 3.32
2.50
-0.00
O
F
O
O
3e
1.93 0.92 0.97 0.94 0.97 1.86
1.00
1.01 1.01
8500 8000 7500 7000 6500 6000 5500 5000 4500 4000 3500 3000 2500 2000 1500 1000 500 0 -500
.0 9.5 9.0 8.5 8.0 7.5 7.0 6.5 6.0 5.5 5.0 4.5 4.0 3.5 3.0 2.5 2.0 1.5 1.0 0.5 0.0 -0.5 -1.0
f1 (ppm)

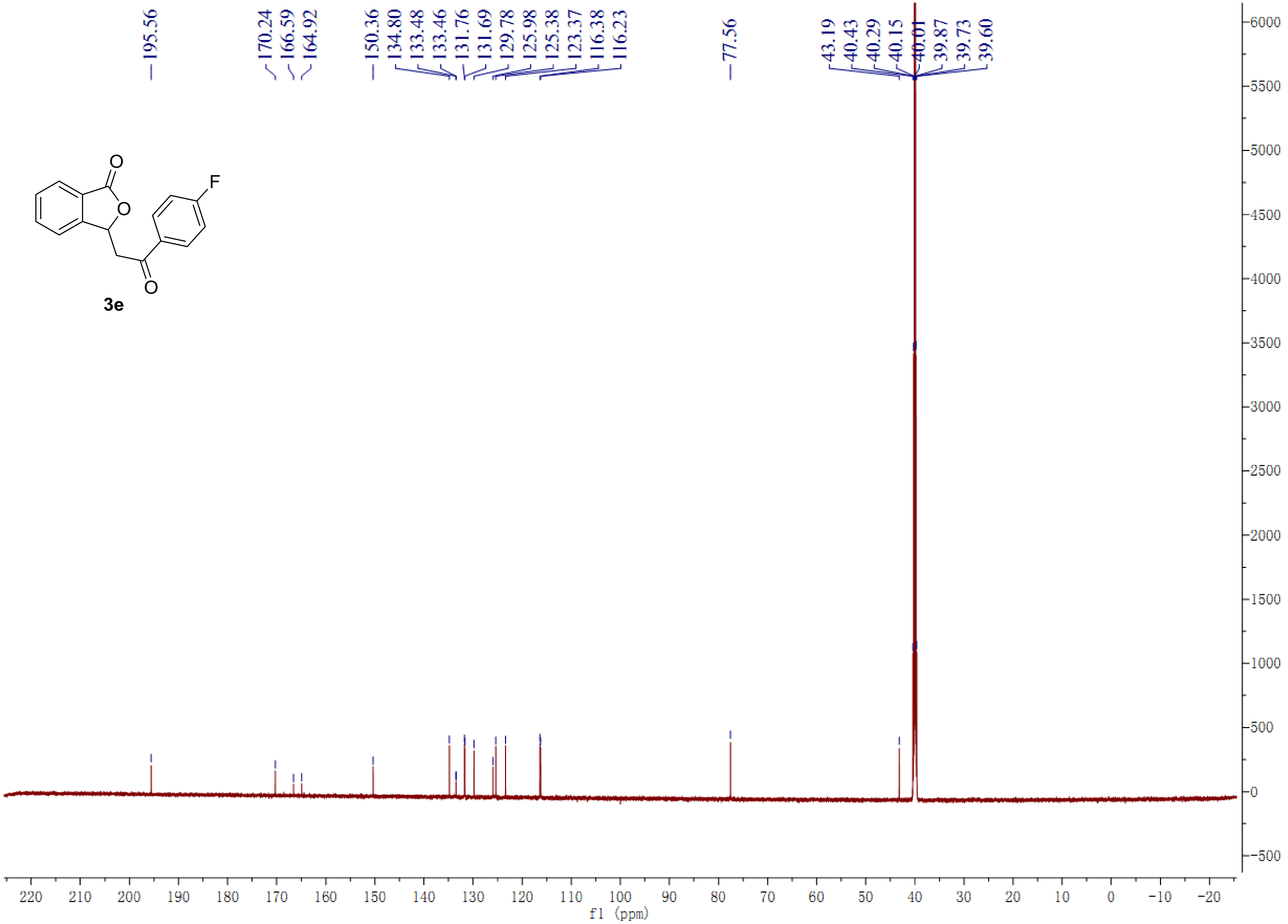
195.56
170.24
166.59
164.92
150.36
134.80
133.48
133.46
131.76
131.69
129.78
125.98
125.38
123.37
116.38
116.23
77.56
43.19
40.43
40.29
40.15
40.01
39.87
39.73
39.60
O
O
F
O
f1 (ppm)

3e

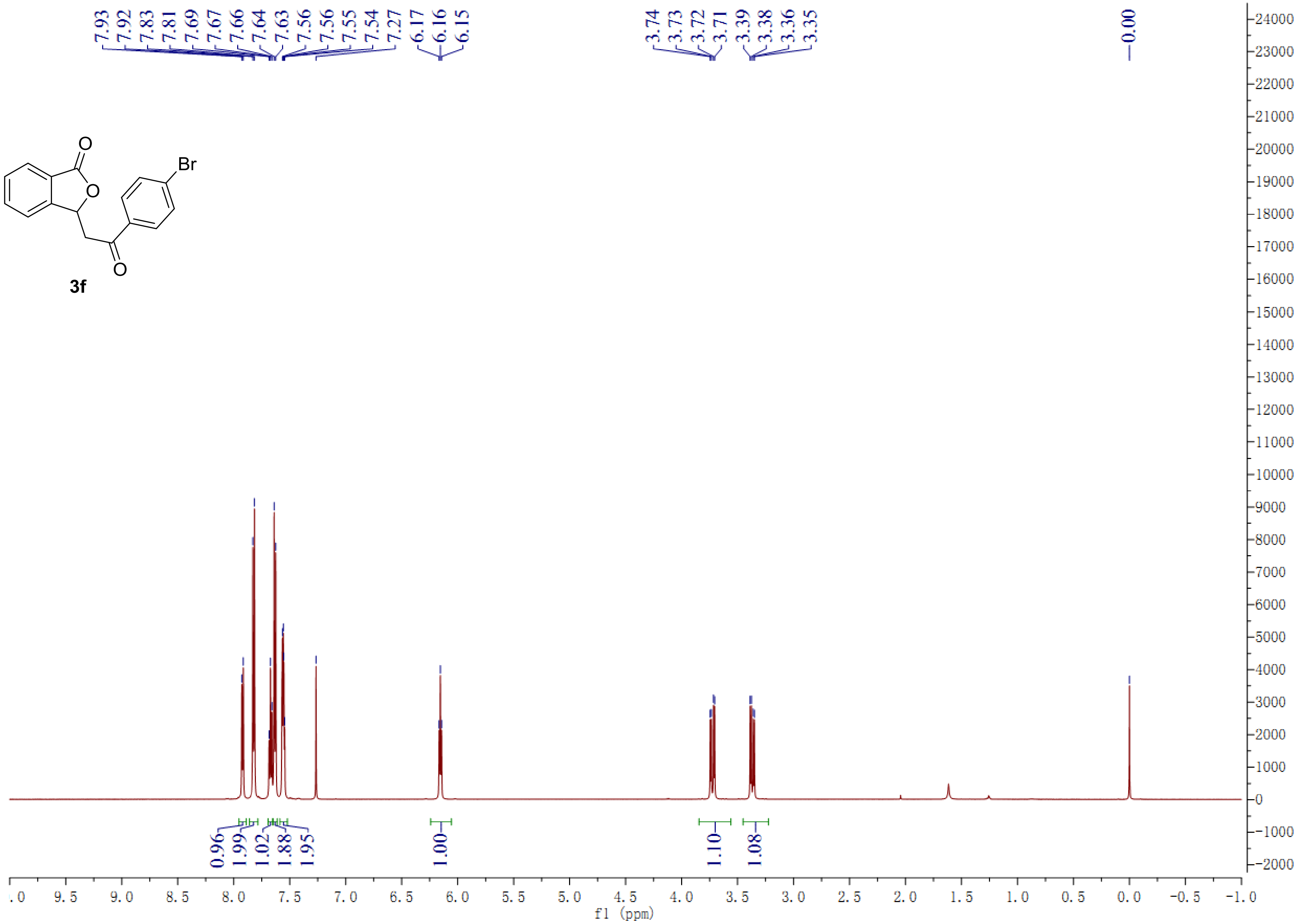
7.93
7.92
7.83
7.81
7.69
7.67
7.66
7.64
7.63
7.56
7.56
7.55
7.54
7.27
6.17
6.16
6.15
3.74
3.73
3.72
3.71
3.39
3.38
3.36
3.35
0.00
Br
O
O
O
3f
0.96
1.99
1.02
1.88
1.95
1.00
1.10
1.08
f1 (ppm)

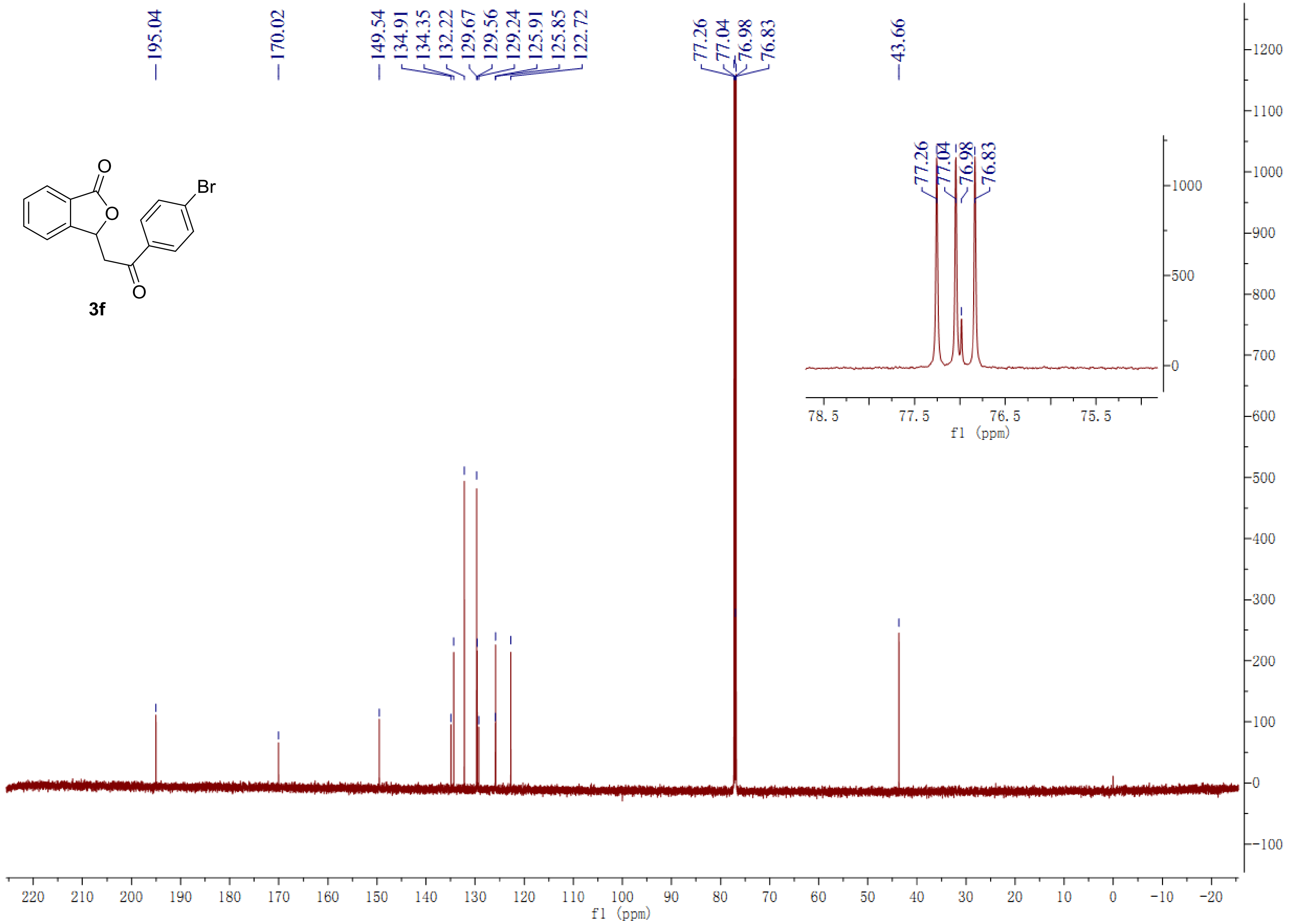
195.04
170.02
149.54
134.91
134.35
132.22
129.67
129.56
129.24
125.91
125.85
122.72
77.26
77.04
76.98
76.83
43.66
O
O
Br
O
3f
f1 (ppm)

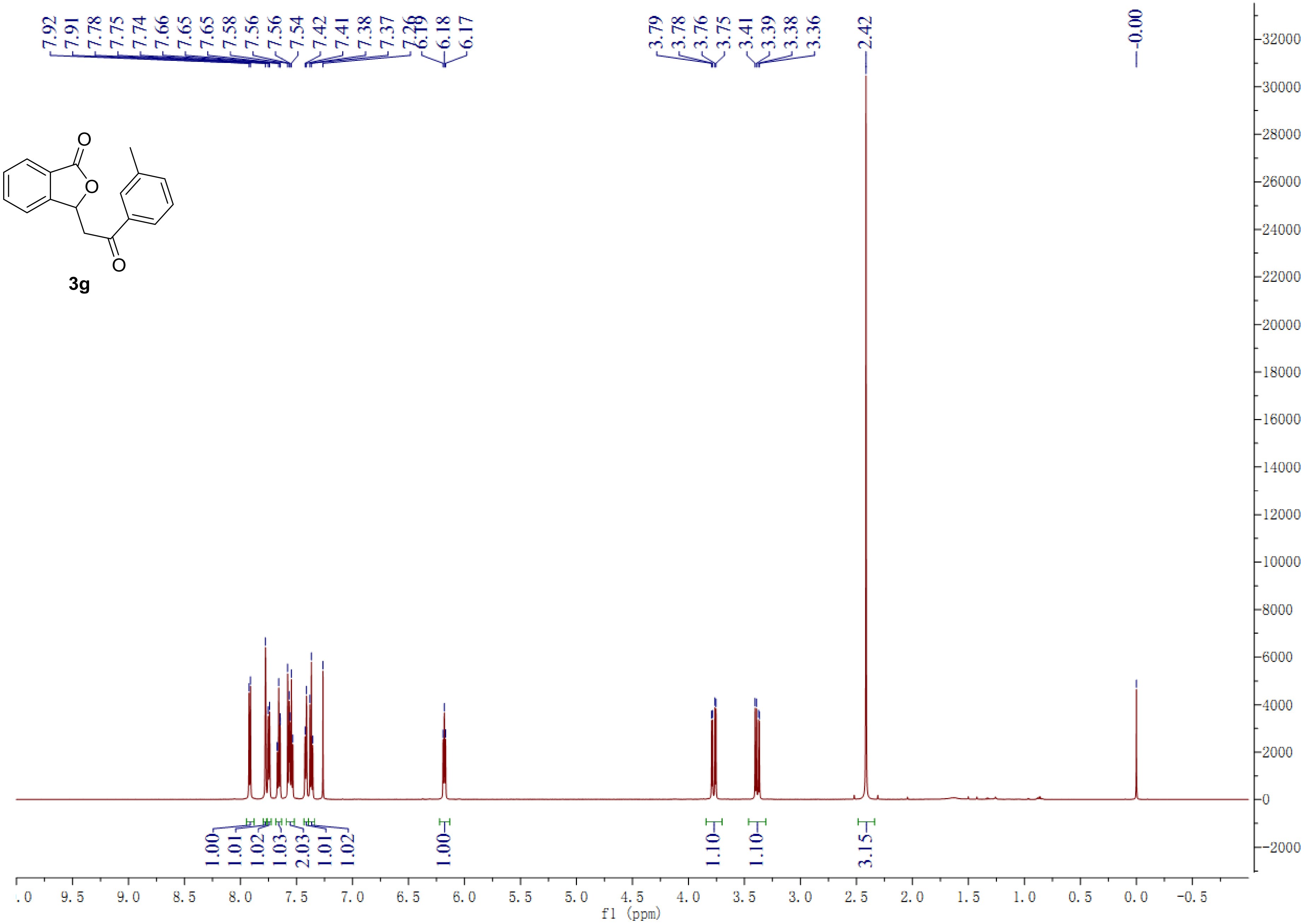

7.92 7.91 7.78 7.75 7.74 7.66 7.65 7.65 7.58 7.56 7.56 7.54 7.42 7.41 7.38 7.37 7.26 6.19 6.18 6.17
3.79 3.78 3.76 3.75 3.41 3.39 3.38 3.36
2.42
0.00
1.00 1.01 1.02 1.03 2.03 1.01 1.02
1.00
1.10
1.10
3.15
32000 30000 28000 26000 24000 22000 20000 18000 16000 14000 12000 10000 8000 6000 4000 2000 0 -2000
.0 9.5 9.0 8.5 8.0 7.5 7.0 6.5 6.0 5.5 5.0 4.5 4.0 3.5 3.0 2.5 2.0 1.5 1.0 0.5 0.0 -0.5
f1 (ppm)
3g

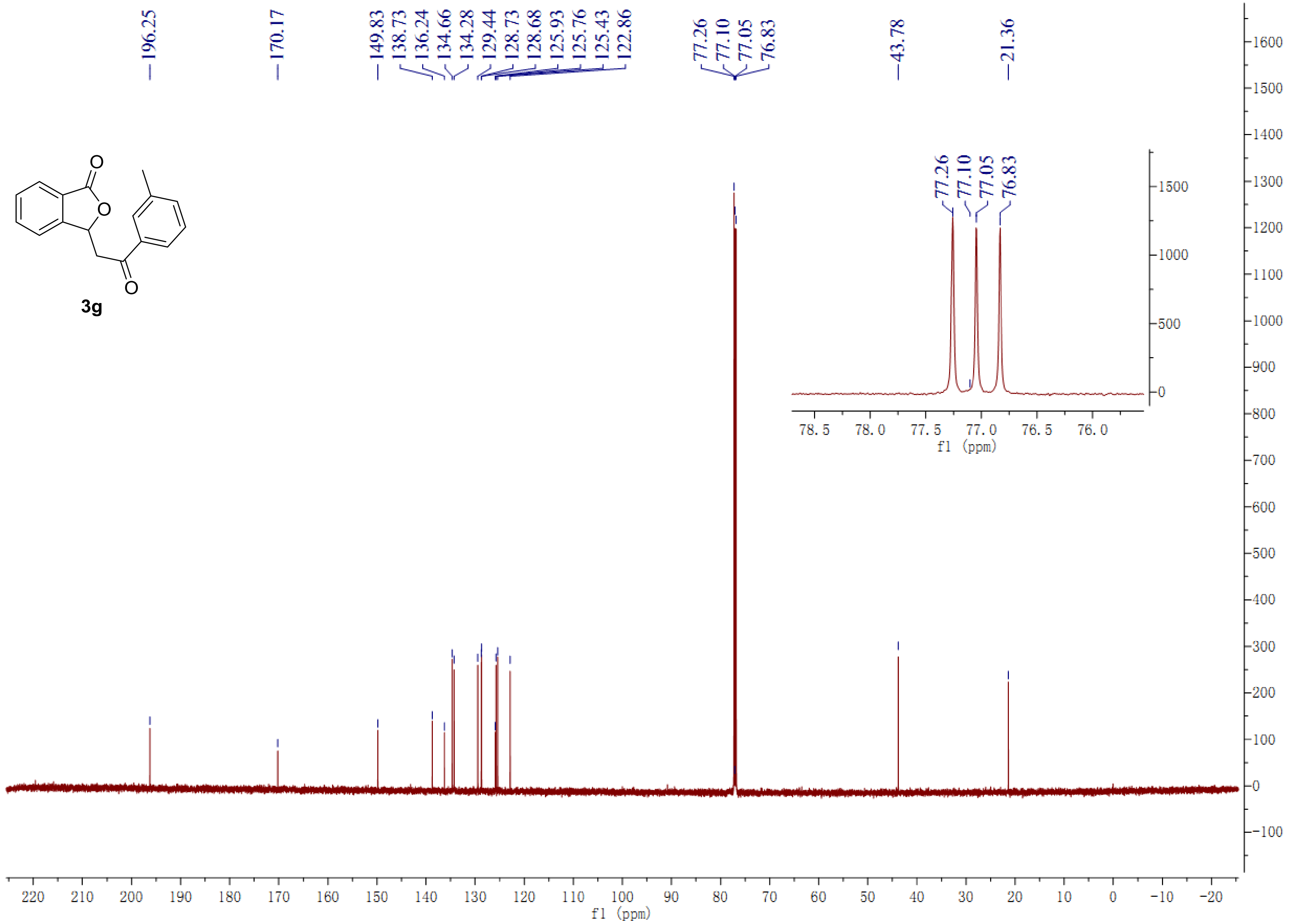

196.25
170.17
149.83
138.73
136.24
134.66
134.28
129.44
128.73
128.68
125.93
125.76
125.43
122.86
77.26
77.10
77.05
76.83
43.78
21.36
3g
f1 (ppm)

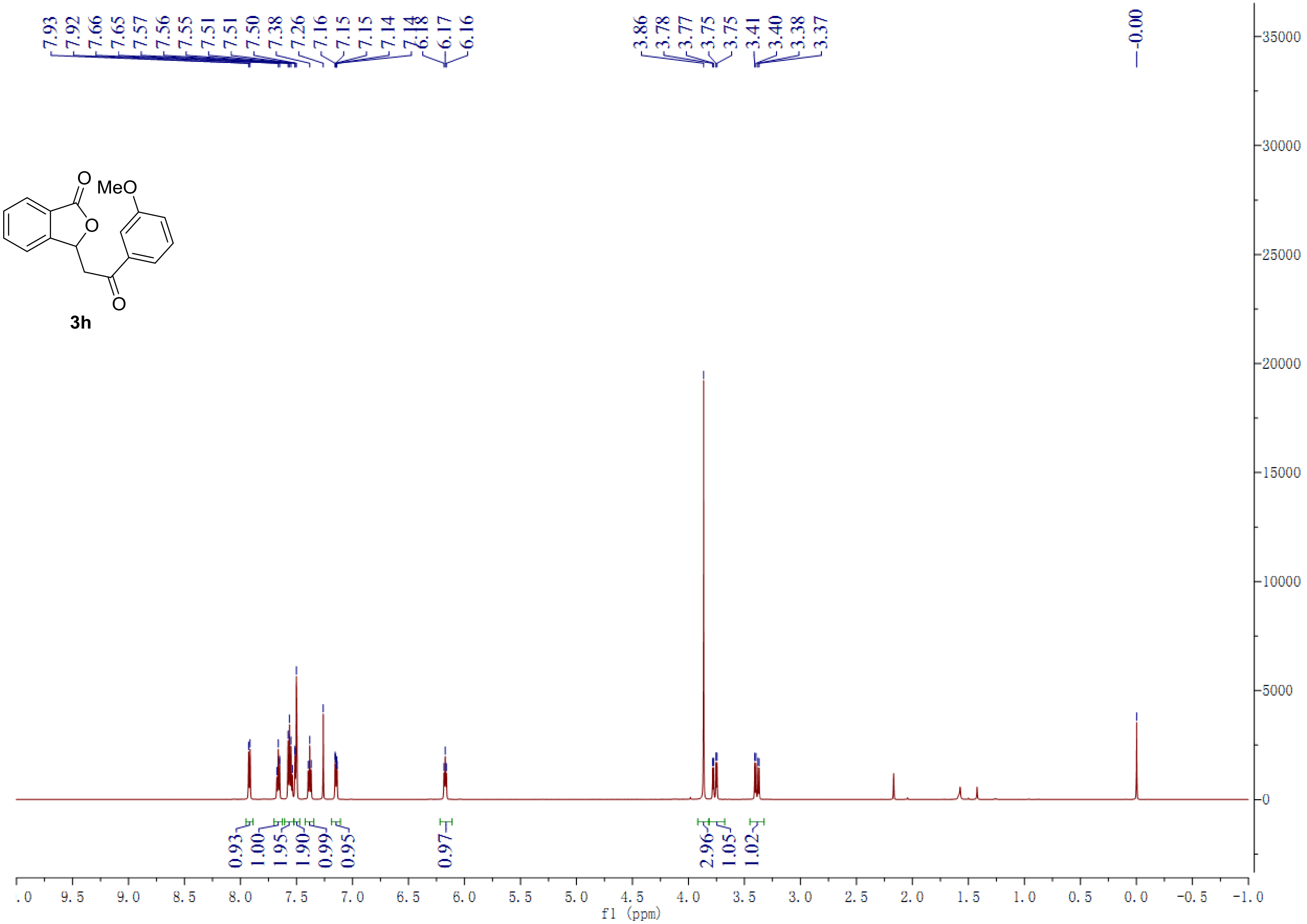
7.93
7.92
7.66
7.65
7.57
7.56
7.55
7.51
7.51
7.50
7.38
7.26
7.16
7.15
7.15
7.14
7.14
6.18
6.17
6.16
3.86
3.78
3.77
3.75
3.75
3.41
3.40
3.38
3.37
-0.00
MeO
O
O
O
3h
0.93
1.00
1.95
1.90
0.99
0.95
0.97
2.96
1.05
1.02
f1 (ppm)

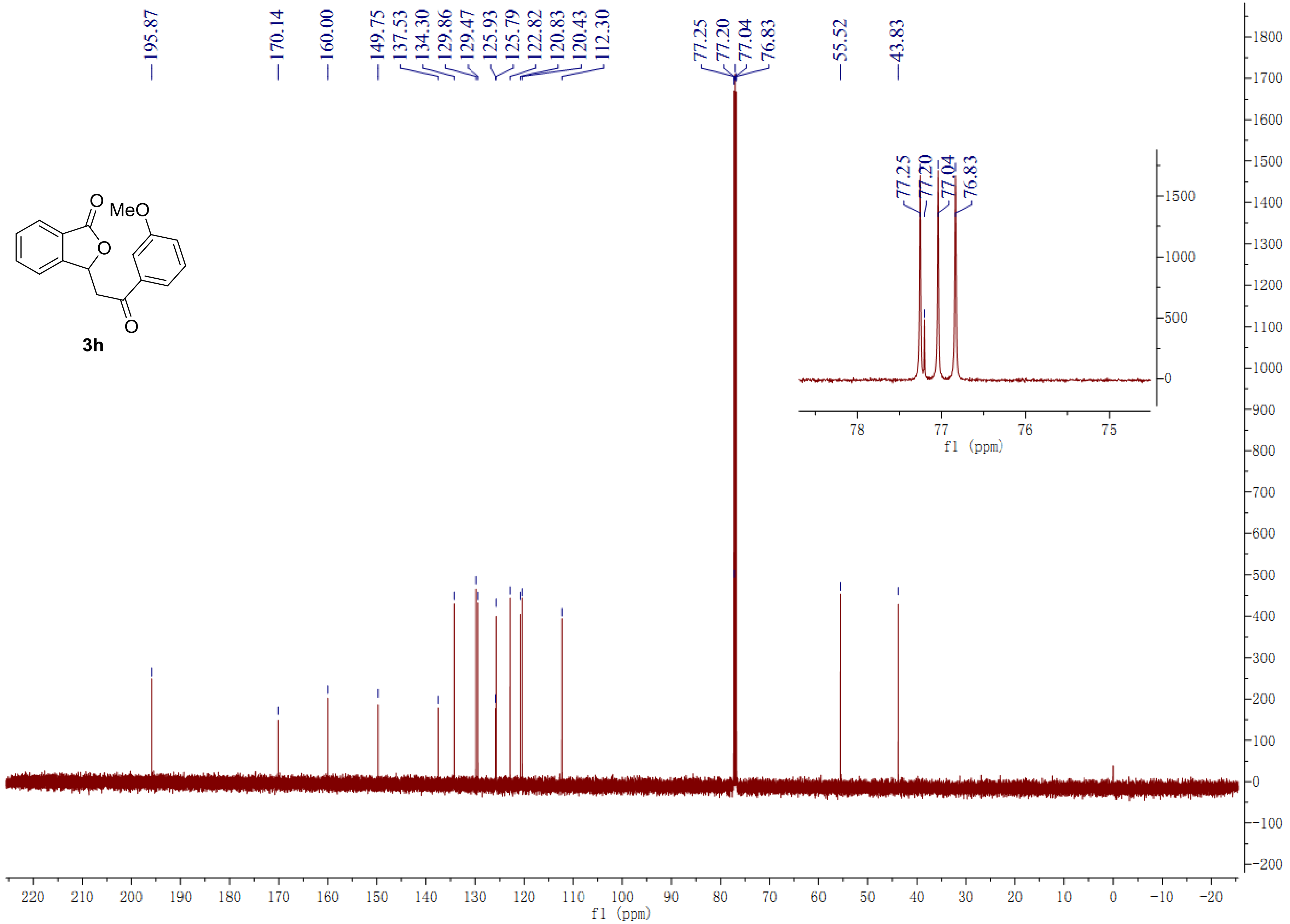

3h

195.87
170.14
160.00
149.75
137.53
134.30
129.86
129.47
125.93
125.79
122.82
120.83
120.43
112.30
77.25
77.20
77.04
76.83
55.52
43.83
f1 (ppm)

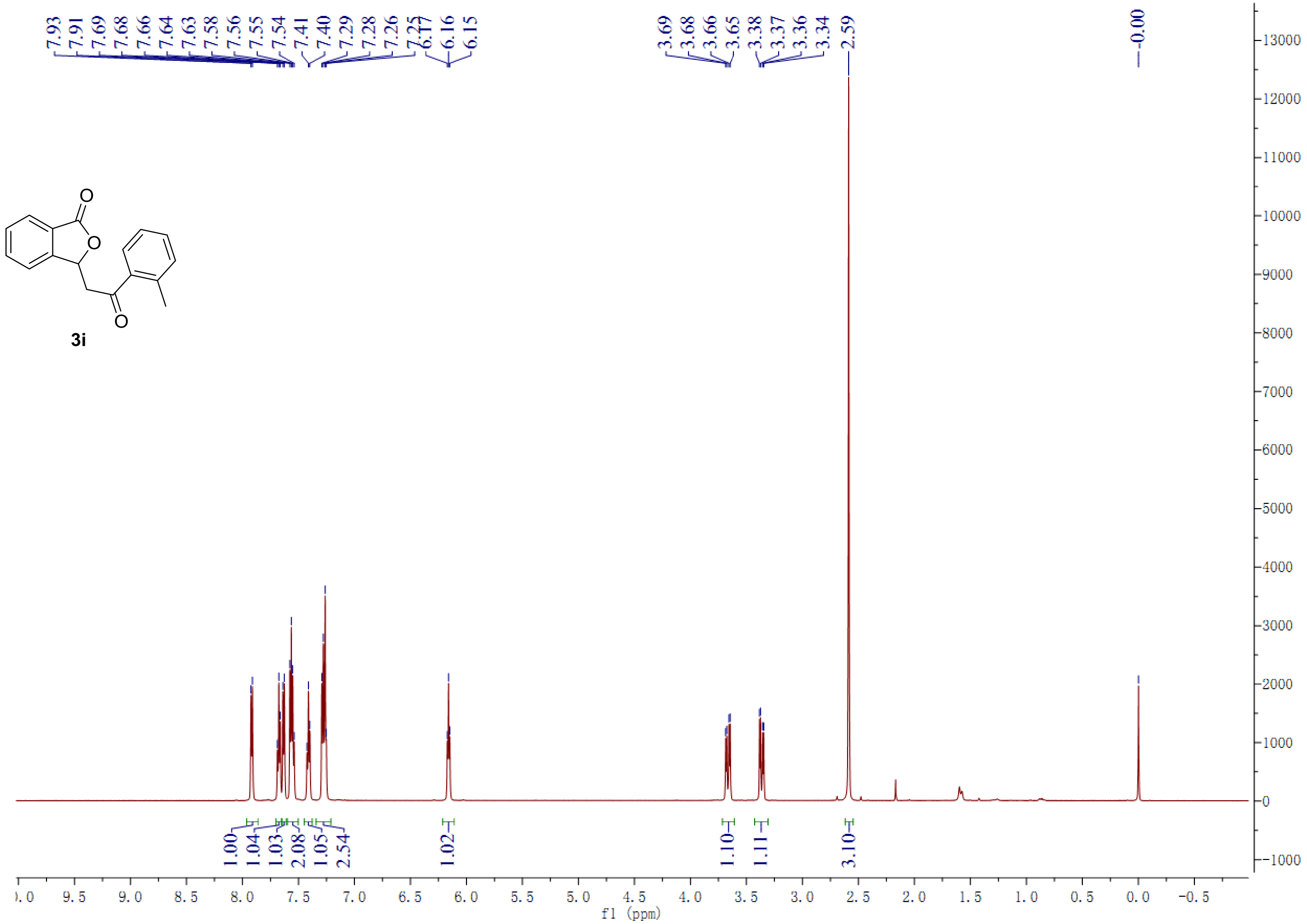
7.93
7.91
7.69
7.68
7.66
7.64
7.63
7.58
7.56
7.55
7.54
7.41
7.40
7.29
7.28
7.26
7.25
6.17
6.16
6.15
3.69
3.68
3.66
3.65
3.38
3.37
3.36
3.34
2.59
-0.00
3i
1.00
1.04
1.03
2.08
1.05
2.54
1.02
1.10
1.11
3.10
f1 (ppm)

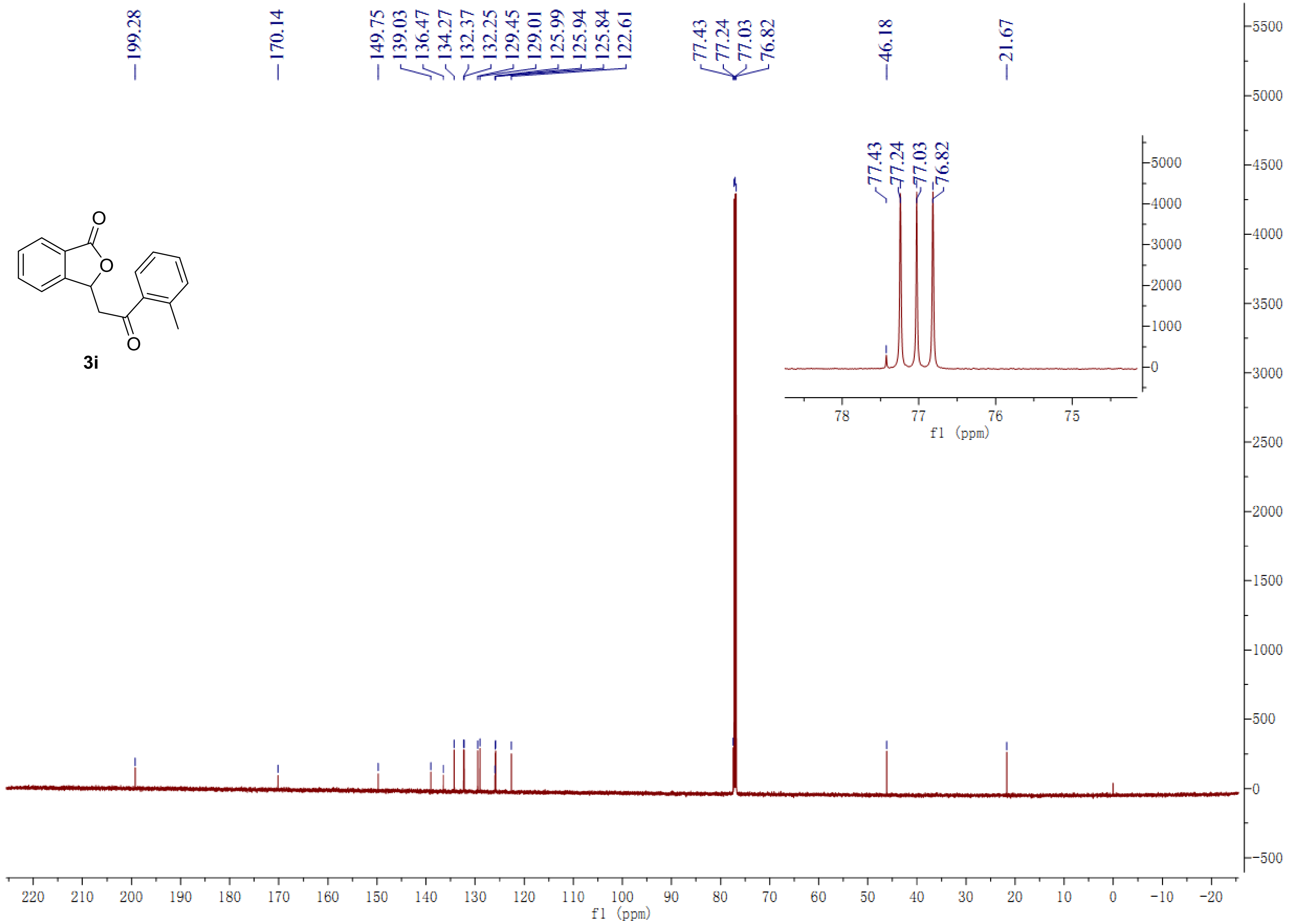
O
O
O
3i
199.28
170.14
149.75
139.03
136.47
134.27
132.37
132.25
129.45
129.01
125.99
125.94
125.84
122.61
77.43
77.24
77.03
76.82
46.18
21.67
f1 (ppm)
220 210 200 190 180 170 160 150 140 130 120 110 100 90 80 70 60 50 40 30 20 10 0 -10 -20
5500 5000 4500 4000 3500 3000 2500 2000 1500 1000 500 0 -500
77.43
77.24
77.03
76.82
78 77 76 75
f1 (ppm)
5000 4000 3000 2000 1000 0

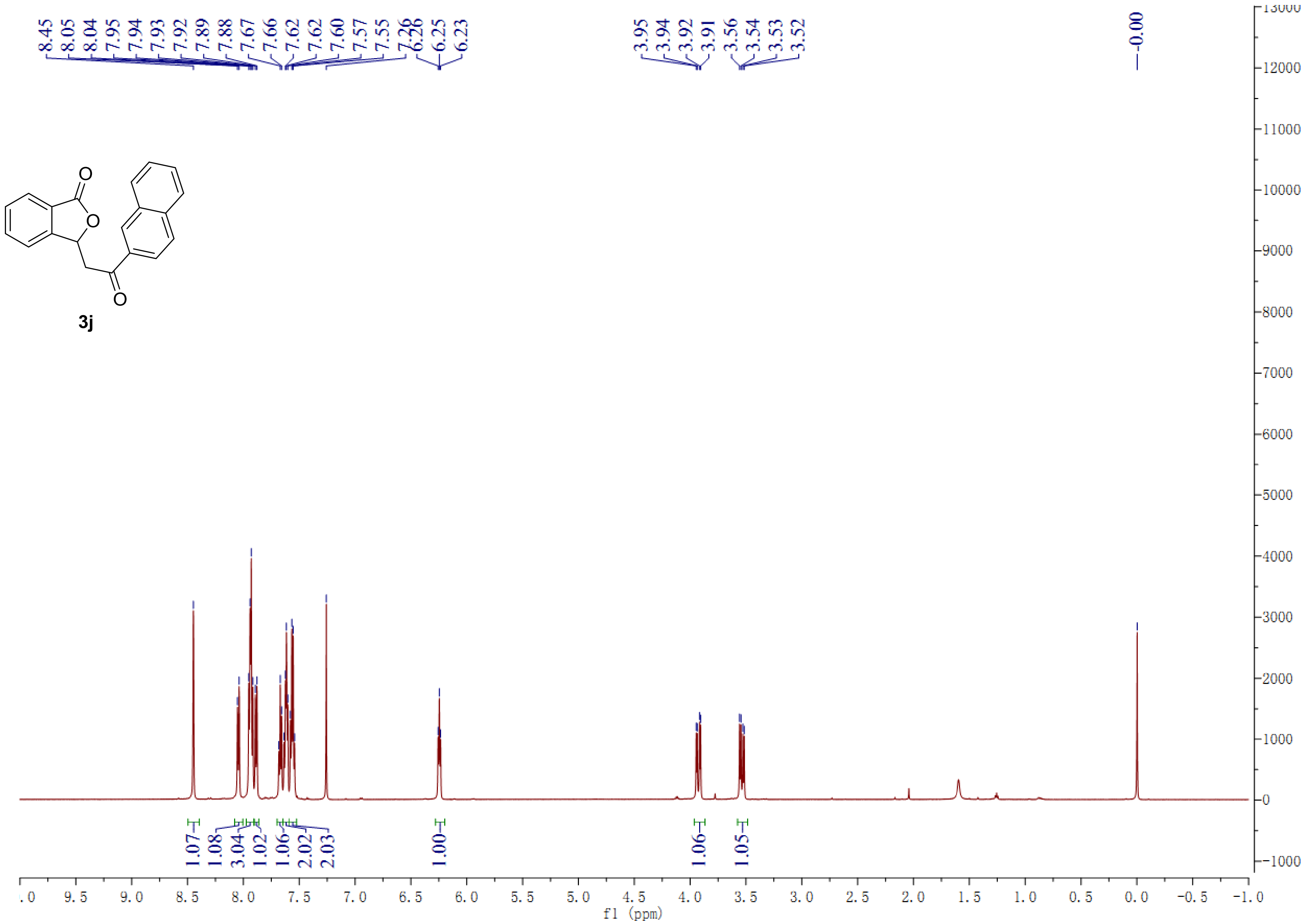
8.45
8.05
8.04
7.95
7.94
7.93
7.92
7.89
7.88
7.67
7.66
7.62
7.62
7.60
7.57
7.55
7.26
7.26
6.25
6.23
3.95
3.94
3.92
3.91
3.56
3.54
3.53
3.52
-0.00
1.07
1.08
3.04
1.02
1.06
2.02
2.03
1.00
1.06
1.05
f1 (ppm)

3j

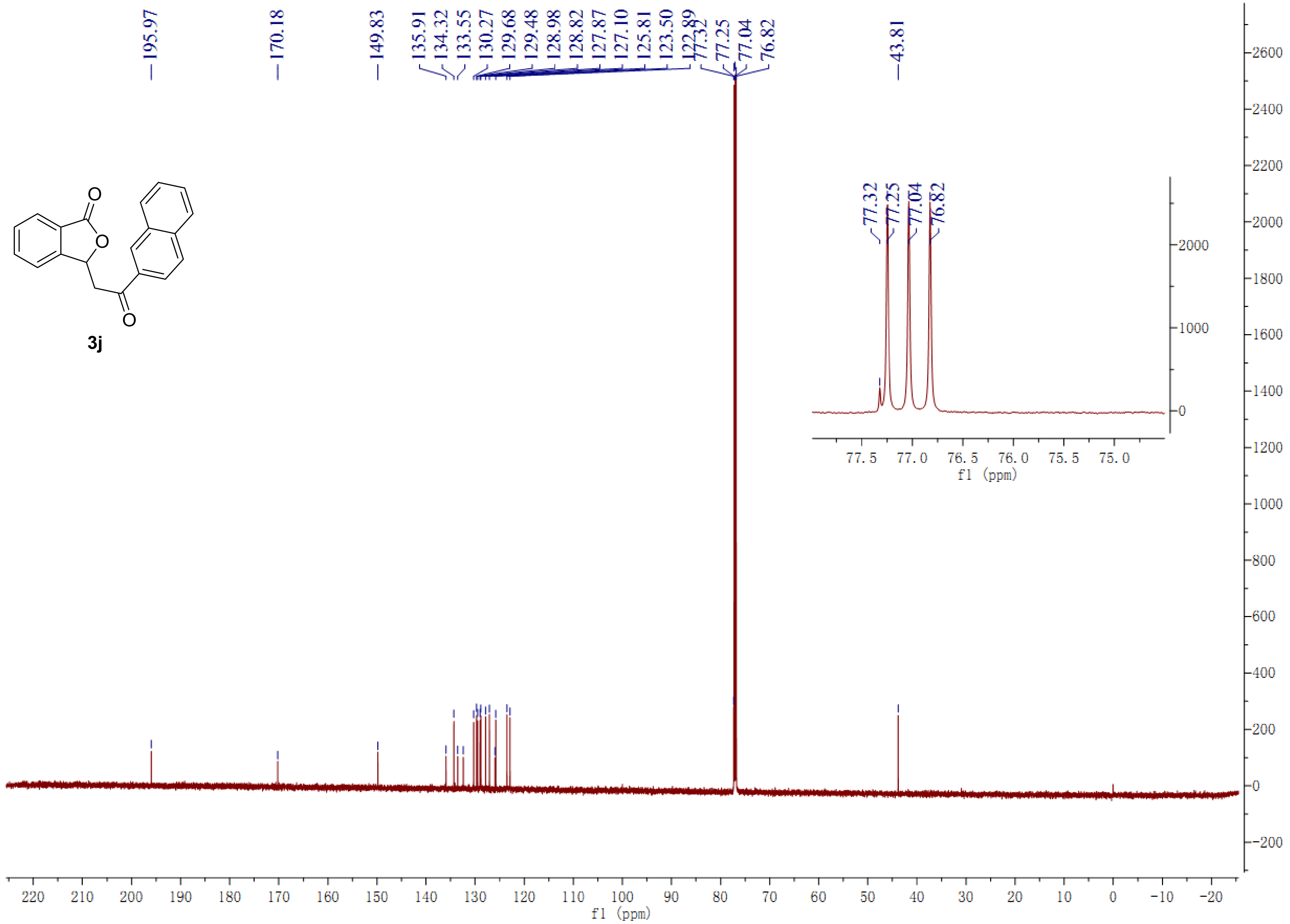
195.97
170.18
149.83
135.91
134.32
133.55
130.27
129.68
129.48
128.98
128.82
127.87
127.10
125.81
123.50
122.89
77.32
77.25
77.04
76.82
43.81
3j
77.32
77.25
77.04
76.82
77.5
77.0
76.5
76.0
75.5
75.0
f1 (ppm)
2000
1000
0
2600
2400
2200
2000
1800
1600
1400
1200
1000
800
600
400
200
0
-200
220
210
200
190
180
170
160
150
140
130
120
110
100
90
80
70
60
50
40
30
20
10
0
-10
-20
f1 (ppm)

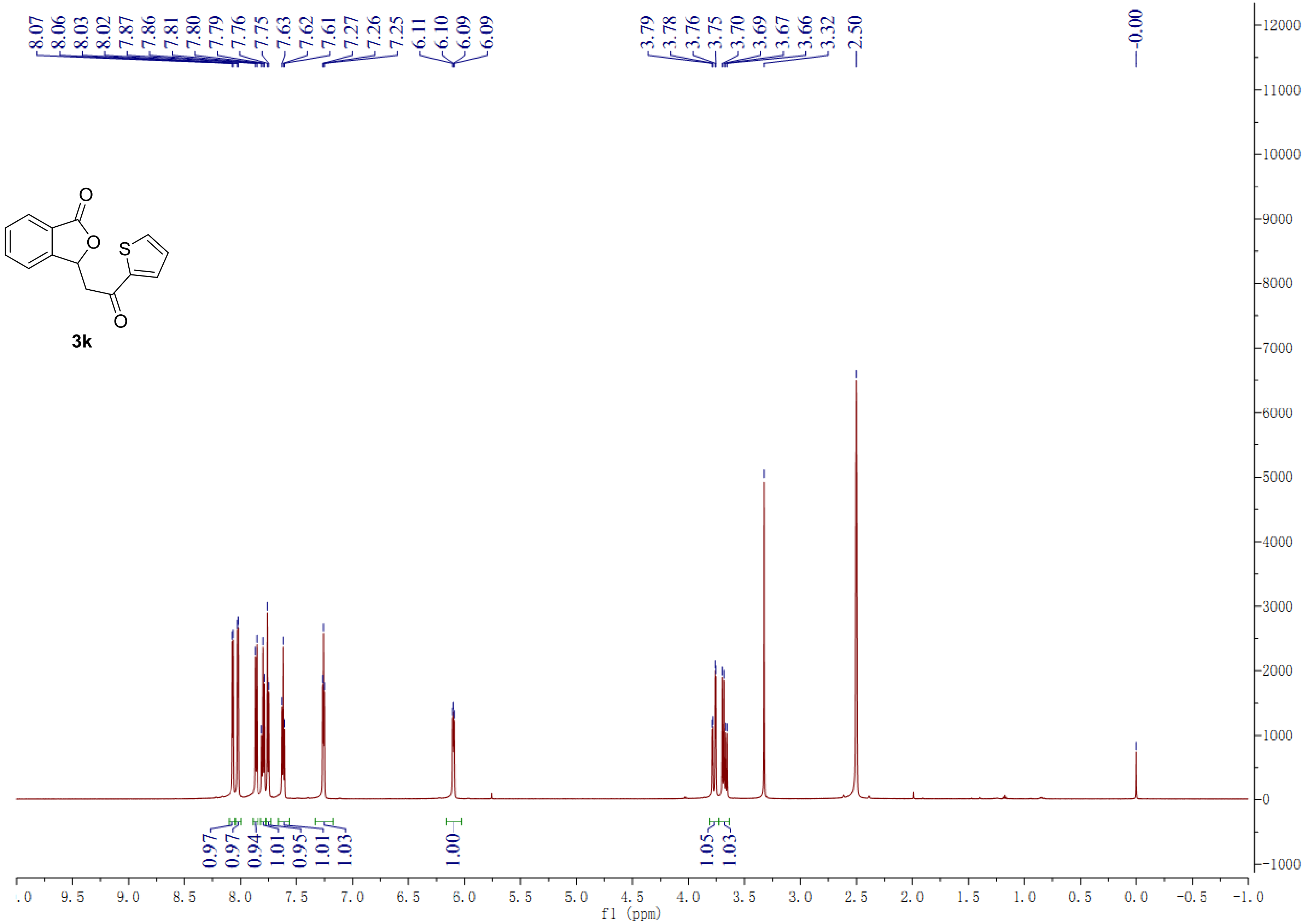
8.07 8.06 8.03 8.02 7.87 7.86 7.81 7.80 7.79 7.76 7.75 7.63 7.62 7.61 7.27 7.26 7.25 6.11 6.10 6.09 6.09
3.79 3.78 3.76 3.75 3.70 3.69 3.67 3.66 3.32 2.50
-0.00
0.97 0.97 0.94 1.01 0.95 1.01 1.03
1.00
1.05 1.03
3k
O
O
S
O
f1 (ppm)
12000 11000 10000 9000 8000 7000 6000 5000 4000 3000 2000 1000 0 -1000
.0 9.5 9.0 8.5 8.0 7.5 7.0 6.5 6.0 5.5 5.0 4.5 4.0 3.5 3.0 2.5 2.0 1.5 1.0 0.5 0.0 -0.5 -1.0

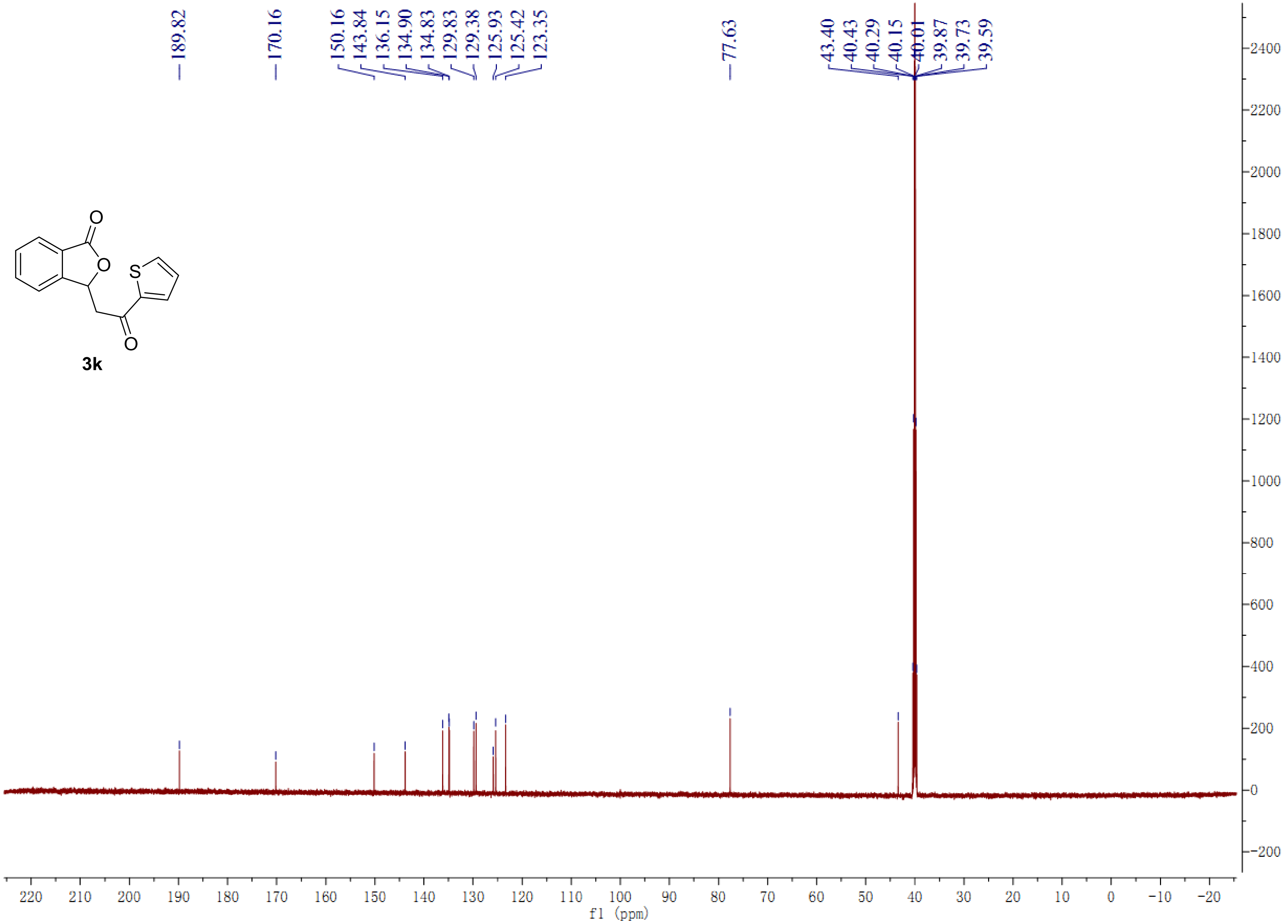
189.82
170.16
150.16
143.84
136.15
134.90
134.83
129.83
129.38
125.93
125.42
123.35
77.63
43.40
40.43
40.29
40.15
40.01
39.87
39.73
39.59
3k
f1 (ppm)

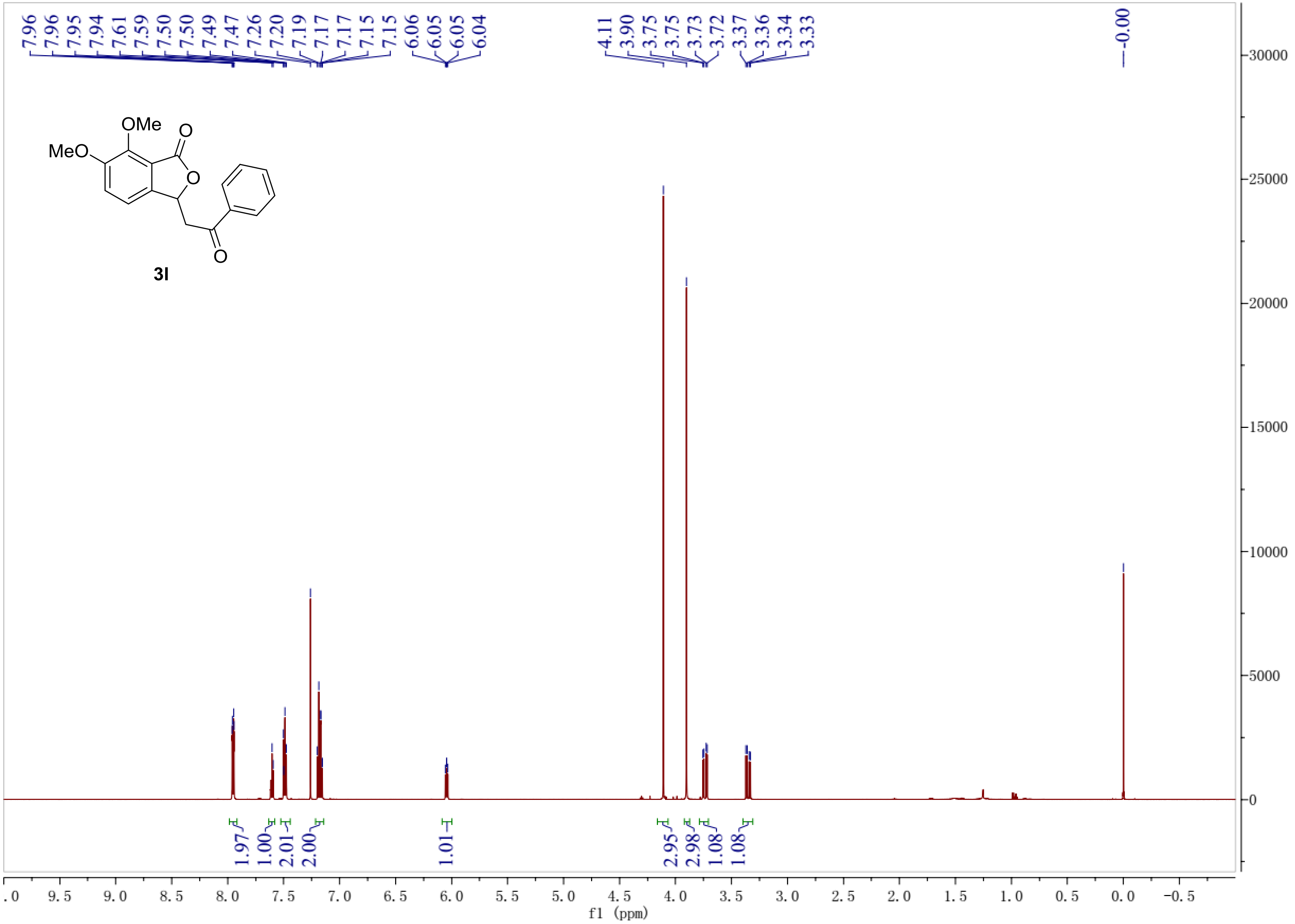

OMe
MeO
O
O
O
3l
7.96
7.96
7.95
7.94
7.61
7.59
7.50
7.50
7.49
7.47
7.26
7.20
7.19
7.17
7.17
7.15
7.15
6.06
6.05
6.05
6.04
4.11
3.90
3.75
3.75
3.73
3.72
3.37
3.36
3.34
3.33
0.00
1.97
1.00
2.01
2.00
1.01
2.95
2.98
1.08
1.08
f1 (ppm)

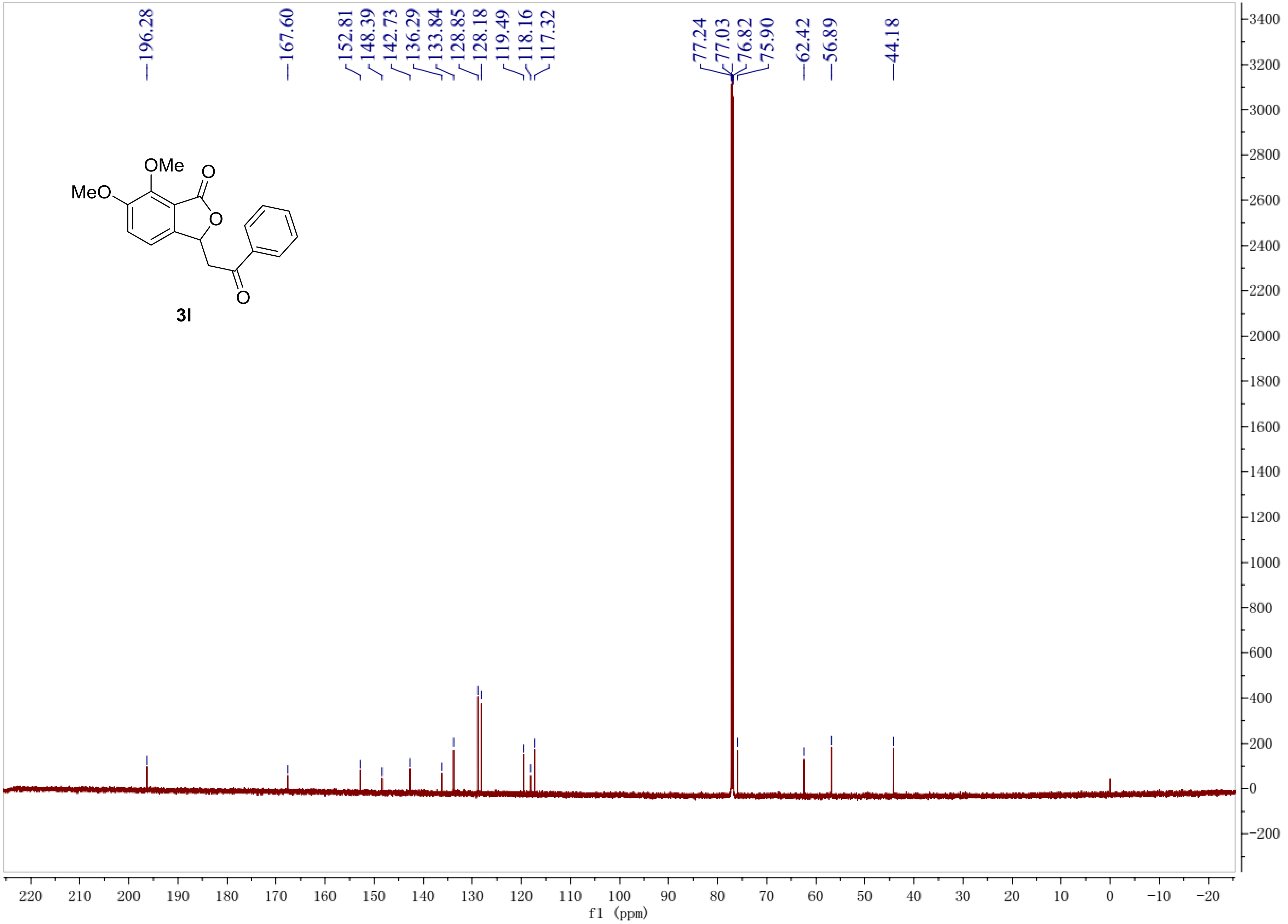

196.28
167.60
152.81
148.39
142.73
136.29
133.84
128.85
128.18
119.49
118.16
117.32
77.24
77.03
76.82
75.90
62.42
56.89
44.18
OMe
MeO
O
O
O
3l
3400
3200
3000
2800
2600
2400
2200
2000
1800
1600
1400
1200
1000
800
600
400
200
0
−200
220 210 200 190 180 170 160 150 140 130 120 110 100 90 80 70 60 50 40 30 20 10 0 −10 −20
f1 (ppm)
